# Age-Specific Income Trends in Europe: The Role of Employment, Wages, and Social Transfers

**DOI:** 10.1007/s11205-021-02838-w

**Published:** 2021-11-30

**Authors:** Bernhard Hammer, Sonja Spitzer, Alexia Prskawetz

**Affiliations:** 1 TU Wien, Institute of Statistics and Mathematical Methods in Economics and Wittgenstein Centre for Demography and Global Human Capital (IIASA, OeAW, University of Vienna), Vienna, Austria; 2 University of Vienna, Department of Demography, Wittgenstein Centre for Demography and Global Human Capital (IIASA, OeAW, University of Vienna), Vienna, Austria

**Keywords:** Generational economy, Income, Intergenerational equity

## Abstract

This study analyses age-specific differences in income trends in nine European countries. Based on data from National Accounts and the European Union Statistics on Income and Living Conditions, we quantify age-specific changes in income between 2008 and 2017 and decompose these changes into employment, wages, and public transfer components. Results show that income of the younger age groups stagnated or declined in most countries since 2008, while income of the older population increased. The decomposition analysis indicates that the main drivers of the diverging trends are higher employment among the older population and a strong increase in public pensions, especially for women.

**Supplementary Information:**

The online version contains supplementary material available at 10.1007/s11205-021-02838-w users.

## Introduction

Income of young adults fell behind income of the older population in most European countries between 2008 and 2017, even in countries where employment rates among the young increased (Eurostat, 2020d, c). Previous research has, however, put little emphasis on the extent and the causes of age-specific differences in income trends. The limited evidence focuses on equivalised household income and shows that income of households with children and young adults stagnated or decreased in most of Europe after the financial crisis, while it increased for the older population (Chen et al., 2018; Wittgenstein Centre, 2020). While employment is identified as an important explanation of the age-specific changes in income, other factors have rarely been studied directly. Chen et al. (2018) suggests that beside employment, public transfers are important drivers of generational disparities in income.

Knowledge about age-specific income trends and their determinants is necessary to understand central economic and demographic developments. After the financial crisis, poverty rates increased or stagnated at high level among young adults and family households in many countries. By contrast, poverty rates among older adults declined during the same period (Eurostat, 2020b). Such developments require attention, because the economic situation of young adults is a crucial determinant of whether and when they form a family, and the number of children they have. Consequently, the deteriorating economic situation of young adults is considered an important factor for the fertility declines in several European countries (Matysiak et al., 2020; IMF, 2018; Goldstein et al., 2013). In addition, most European countries experienced a baby boom in the decades after the second world war, with varying degree and length. The baby-boomer are currently entering retirement or will reach retirement age in the next two decades, which will increase old-age related spending (European Commission, 2018, 2021) and the demand for higher taxes from the working age population. The COVID-19 pandemic will further increase the pressure on young generations in the years to come. It is thus of utmost importance to study the drivers of income changes at each life-stage, to understand their relation with social, economic and demographic developments.

Employment is particularly important for the understanding of age-specific differences in income trends, most notably in Southern Europe. Between 2008 and 2017, employment rates of the 15–39-year-old decreased by 9 percentage-points in Italy, Greece, and Spain, while employment among the population aged 40–59 increased (Eurostat, 2020c). In addition, young people find themselves increasingly entrapped in insecure and temporary work (Barbieri et al., 2019; Garibaldi and Taddei, 2013). This strong deterioration of employment opportunities for the young in Southern Europe is found to be reinforced by labour market institutions that generate a pronounced duality in the labour markets: the older, permanent employees are strongly protected, while the insecurity due to a flexibilisation of the labour market has been entirely placed on young cohorts (Barbieri, 2011; Chauvel and Schröder, 2014). Employment of the young also decreased in many Western and Northern European countries, however, to a much lesser degree than in Southern Europe. By contrast, in most Central and Eastern European (CEE) countries employment increased for young adults, and even more so for older working age adults (Eurostat, 2020c). Aliaj et al. (2016) show that employment among the population 50+ increased in Belgium, France, Germany, and the Netherlands in the last decades uninterrupted by the financial crisis, but they do observe a reduction in average hours worked. The change of employment rates in percentage points was more favourable for the population 40–59, compared to the population 15–39, in all European countries except the three Baltic countries, Iceland, and Switzerland. Changes in employment rates differ not only by age groups, but also by gender. Employment among the older working age population increased much stronger for women, compared to men.

Although employment is an important driver of income trends, it does not entirely explain the unequal developments in income across ages. For example, equivalised income of the population aged 65+ increased by five per cent in Italy and seven per cent in Spain, yet without associated increases in employment (Eurostat 2020d). This suggests that in addition to employment, changes in wages and public transfers are important components to consider when analysing the underlying causes of diverging income trends across age groups. Chen et al. (2018), for example, suggests that in addition to labour market developments, the design of social protection is an important dimension to consider, since it potentially guards the income of older persons while offering little assistance to young individuals.

Despite the important implications of age-specific income trends for societal and demographic development, no previous research, to our knowledge, has systematically analysed these changes in a comparative manner for Europe. Moreover, it remains unclear how employment, wages, and public transfers contribute to changes in income across different generations in the previous decades. Our study aims at closing these gaps by addressing the following research questions based on data from the European system of Accounts (ESA) and the European Statistics on Income and Living Conditions (EU-SILC):

1. How did aggregate per-capita income and its components change between 2008 and 2017?
2. How do these changes differ between age groups?
3. What drives age-specific income trends: changes in employment, wages, or transfer income?

The countries included in the analysis are Austria, Estonia, Greece, France, Italy, Poland, Slovenia, Spain, and Sweden. EU-SILC provides comparable information on individual gross income and on household net income in all EU countries. The nine countries included in our analysis also provide information on individual net income, i.e. on net income from employment, self-employment, and net public benefits. We focus on individual income rather than household income, since the latter potentially hides age-specific changes when various generations live in the same household.

Moreover, the set of countries allows us to study different welfare regimes and various economic developments during the observed period. In the presentation of the results, we group countries based on their developments in aggregate and age-specific income. In particular, we group the Southern European countries together (Italy, Greece and Spain), the Western and Central European countries (Austria, France and Slovenia), and the Eastern and Northern European countries (Poland, Estonia and Sweden). The development in aggregate and age-specific income was similar within these groups of countries, while it differed considerably across these country-groups.

Our paper is divided into three parts, in line with our three research questions. First, we measure total changes in income and public redistribution between 2008 and 2017, using aggregate economic ESA data. Aggregate data are more comprehensive and reliable than survey-based data and thus constitute a benchmark for comparing and verifying the survey-based results. Second, we analyse age-specific changes in income between 2008 and 2017, using micro-level EU-SILC data. In the third part of the paper, we decompose the age-specific changes in income into its main components, thereby identifying how changes in employment, wages and social benefits affect age-specific income trends.

## Data and Methodology

### Aggregate Income in the European System of Accounts

Data from ESA provide detailed information on the level and type of household sector income as well as on public redistribution (for a detailed description of ESA see European Commission, 2013). The annual sector accounts are available from the Eurostat database (Eurostat, 2020a). Our focus is on four quantities: (1) primary income before public redistribution, (2) payments of taxes and social contributions, (3) receipt of social benefits, and (4) disposable income. Taken together, these four quantities summarize changes in total income, the extent of public redistribution, and its consequence for disposable income. For comparison, we also consider GDP per capita and its changes, because it is the most common measure of economic development.

1. *Primary income* in ESA measures income that is generated by direct participation in the production process, before any payment of taxes or social contributions. The largest component of primary income of the household sector is the compensation of employees, which consists of all types of remuneration for work, including social contributions paid by employers. Furthermore, primary income includes asset income, such as interest, dividends, the return to owners of unincorporated enterprises and owner-occupied housing.
2. The *income tax ratio* measures income taxes relative to primary income and thus serves as an indicator of the total size of public redistribution. For calculating the income tax ratio, we combine several quantities from ESA and information on the tax structure (European Commission, 2019). In particular, our definition of the tax ratio takes into account taxes on production, taxes on income and wealth, as well as social contributions. Taxes paid on public transfers such as pensions are not included, because national variations in tax systems could bias cross-country comparisons. The concept differs from the more common tax-to-GDP ratio, since our goal is not the measurement of the total tax burden, but to capture all direct taxes that are paid out of primary income. Consequently, taxes on products are not included in the income tax ratio, because they constitute part of consumption expenditure and affect disposable income only indirectly.
3. The *benefit ratio* is calculated as the ratio of total cash benefits from the government to primary income of households. It serves as an indicator of the importance of public redistribution via cash transfers. In ESA, the largest type of benefits are social benefits, including pensions, unemployment benefits, family allowances and other types of social benefits. To be consistent with our definition of the tax ratio, we measure benefits net of taxes and social contributions that are paid on these benefits. In addition, the benefit ratio includes other current transfers from the government sector to households.
4. *Disposable income* is an indicator of economic wellbeing of households. It is calculated as primary income less taxes plus cash benefits and represents the income of households that can be used for consumption and saving.

### Age-Specific Income in EU-SILC

The age-specific analysis of income changes is based on EU-SILC data. We distinguish three age-groups: the young working age population at age 20–39, the older working age population at age 40–59, and the elderly population aged 60+. We opted for 20-year age groups to have sufficient observations for reliable estimates when distinguishing also by type of income and gender. Ages 20–39 coincide with the life stage of early labour market career, family formation and childbearing. The age group 40–59 consists mainly of persons that made their family decisions already, who do not have care responsibilities for small children anymore, and who are active on the labour market. The age border of 60 corresponds to actual retirement age in most countries; thus, the age-group 60+ captures therefore mainly the group of retirees. For each of the three age groups, we calculate the changes in age-specific mean income in real terms between 2008 and 2017. Means are required by the decomposition analysis we apply to individual income as described below. However, we also provide estimates of changes in median income as well as the changes in the first and the third quartile.

All income components are assigned to individuals, also components that are given at household level in EU-SILC, including family benefits, imputed rents, asset income and income of persons younger than 16. The details of allocating household level income to individuals are given in “Allocation of Household-Level Income to Individuals” in the Appendix. Sensitivity analysis has indicated that the assumptions for the distribution of household level income have little effect on the level and change of age-specific income. First, income at household level accounts for less than 20 percent of total income, with imputed rents as largest component. Second, the age group of recipients of household-level income can be identified unambiguously in most households. The same is true for gender-specific results except for Poland, where an increase in the monetary support of families resulted in an increase of income among the 20–39-year-old. Hence, the gender-specific results for Poland depend on how these increased benefits are allocated within a couple.

Age-specific changes over time are often analysed using an age-period-cohort (APC) approach. An APC approach distinguishes *period effects* that influence income earners at all ages and from all birth cohorts, *age-effects* that comprise changes in fundamental life-cycle patterns, and *cohort-effects* that only affect persons born in given years, such as an economic shock that affects all persons newly entering the labour market (De Fraja et al., 2017; Mroz and Savage, 2006). Since the three APC dimensions are perfectly collinear, it is not possible to unambiguously disentangle the distinct effects. Although previous literature has suggested methods to overcome this identification issue, their applications are frequently disputed and their results often unintuitive (Bell, 2020; Fosse and Winship, 2019). The difficulty to distinguish between cohort and age-effects is aggravated by the short time span covered by EU-SILC, which only provides suitable pseudo-panel data for a period of ten years. Since economic shocks, such as the financial crisis or the sovereign debt crisis, affected consecutive birth cohorts in a similar way, it is currently not possible to identify if the effect is indeed short-lived and affects only few cohorts, or if it resulted in a lasting change of the age patterns. We thus refrain from decomposing APC effects, acknowledging that the difference in income trends between age groups reflects both, age and cohort effects.

### Decomposition of Income Changes

Changes in mean income between 2008 and 2017 are decomposed to identify the role of employment, wages, and public transfers. This analysis is solely based on income and employment data in EU-SILC. We distinguish between three income components: Per-capita averages of total income (Y) are the sum of average income from employment (YL), public benefits (YB) and other income (YO), which includes for example asset income and inter-household transfers. The sub-components of YO are comparably small, which is the reason for combining them into a single category.

For each age group *i*, we decompose the percentage change in total income between 2008 and 2017 into the sum of the changes of its components, with $\varDelta _\%$ referring to changes in percent of total income in 2008:^1^

1 $$\begin{aligned} \varDelta _\% \text {Y}_i = \varDelta _\% \mathrm {YL}_{i} + \varDelta _\% \text {YB}_{i} + \varDelta _\% \text {YO}_{i} \end{aligned}$$

To identify the effect of changes in employment rates on $\varDelta _\% Y_i$, we further decompose the components $\varDelta _\% \mathrm {YL}_{i}$ and $\varDelta _\% \mathrm {YB}_{i}$, which are both strongly related to employment rates. While employment-income arises exclusively to persons in employment, benefits are directed mainly at the non-employed population, such as pensioners, unemployed persons, or persons on paternal leave. Average income from employment can be written as product of the employment rate ($l_i$) and average income of each employed person ($\text {yl}_i$):

2 $$\begin{aligned} \text {YL}_i = \text {yl}_i*l_i \end{aligned}$$

Likewise, average benefits can be written as product of the share of non-employed persons $(1-l_i)$ and average benefits per non-employed person ($\text {yb}_i$):

3 $$\begin{aligned} \text {YB}_i = \text {yb}_i*(1-l_i) \end{aligned}$$

The total change of $\mathrm {YL}_i$ and $\mathrm {YB}_i$ is allocated to each factor according to its relative change, i.e. if income per employed person increases by 10 percent and the employment rate by 5 percent, we would allocate 2/3 of $\varDelta _\% \text {YL}$ to income per employed person and 1/3 to the employment rate. We can then write the total changes in income as sum of five components:

4 $$\begin{aligned} \varDelta _\% \text {Y}_i = \varDelta _\% \mathrm {l}_{i} + \varDelta _\% \mathrm {yl}_{i} + \varDelta _\% (1-\text {l}_{i}) + \varDelta _\% \text {yb}_{i} + \varDelta _\% \text {YO}_{i} \end{aligned}$$

The method of decomposing $\varDelta _\% \mathrm {YL}_{i}$ and $\varDelta _\% \mathrm {YB}_{i}$ is described in more detail and with an example in “Decomposition of Income Changes” of the Appendix.

## Results

### Changes in Aggregate Income

The financial crisis and the sovereign debt crisis had a much stronger effect on income of the household sector than indicated by changes in GDP. While GDP per capita declined from 2008 to 2017 only in Greece and Italy (Column 1 in Table 1), per capita primary income of households declined in six out of the nine analysed countries (Column 2). The decline was most pronounced in Greece with 32 per cent, and in Italy and Spain, with a decline of more than 10 per cent. Primary income decreased slightly in Austria, France and Slovenia and slightly increased in Poland. Only households in Estonia and Sweden had considerably more income per capita in 2017 than in 2008 with an increase of 16 per cent.

The extent of public redistribution increased in all nine countries during the period 2008–2017. The tax ratio stagnated in Spain and Sweden, and increased in the other countries (Column 3 of Table 1), with the highest increase of 7 per cent in Greece. Part of the higher taxes were used to finance increasing cash transfers; the benefit ratio increased in all analysed countries. This increase of social benefits relative to primary income was most pronounced in Italy with an increase of 5 percentage points, and in France and Spain with an increase of 4 percentage points. The changes in the tax ratio and the benefit ratio measure mainly the redistribution within the household sector, with a small effect on the household sector as a whole. Therefore, changes in disposable income follow largely the changes in primary income. Disposable income of the household sector increased in Estonia, Poland and Sweden, and declined in the other six countries.

**Table 1:** Real income per capita, taxes and benefits and their change during the 2008–2017 period (in 2018 prices). *Source*: Authors’ own calculations based on the ESA 2010 annual sector accounts

Country	(1)	(2)	(3)	(4)	(5)
	GDP per capita	Primary income (PI)	Income tax ratio (% of PI)	Benefit ratio (% of PI)	Disposable income
*2017–Values*					
Austria	42,100	26,638	38	28	23,887
France	34,385	23,008	45	33	20,258
Slovenia	20,819	12,627	35	27	11,572
Greece	16,451	10,498	33	28	9962
Spain	24,971	16,042	32	24	14,818
Italy	28,662	19,637	36	26	17,730
Estonia	18,133	10,537	32	24	9690
Poland	12,309	7,650	30	24	7249
Sweden	48,026	29,426	38	23	25,005

Changes 2008–2017					
	(%)	(%)	(%-points)	(%-points)	(%)
Austria	2	−3	2	2	−2
France	3	−1	4	4	−1
Slovenia	1	−3	1	3	−2
Greece	−24	−32	7	3	−35
Spain	0	−10	0	4	−7
Italy	−7	−14	3	5 ara>	−12
Estonia	15	16	1	1	17
Poland	11	3	1	2	4
Sweden	8	16	0	1	17

### Age-Specific Changes

The analysis of income changes by age reveals large disparities between age groups (Table 2). Income of the 20–39-year-old declined in five of the nine analysed countries and only in Estonia it increased by more than 5 per cent. The income trends were more favourable for the older working age population at age 40–59, where only in Greece, Italy, and Spain income per capita declined. However, the gains in income were concentrated in the population 60+, with absolute gains in all countries except Greece. Even in Greece the decline in income was much less for the population 60+ compared to the prime working age population. Income of the elderly population increased also in Italy and Spain, despite the strong declines in the working age population. It increased strongly in Austria, France and Slovenia, while income of the working age population merely stagnated with a change of 4 percent or less over the whole period from 2008 to 2017.

**Table 2:** Mean individual net income by age *Source*: Authors' own calculation based on EU-SILC cross-section data for 2009 and 2018 (income reference years 2008 and 2017)

Individual net income in 2017				
Country	Age 20–39	Age 40–59	Age 60+	Total (20+)
Austria	21,458	30,028	24,665	25,695
France	21,121	30,726	30,012	27,512
Slovenia	11,345	14,954	11,889	12,839
Greece	6,506	10,661	9,965	9235
Spain	12,225	18,523	17,422	16,326
Italy	12,676	21,089	20,769	18,692
Estonia	11,025	11,879	7,308	10,111
Poland	6,523	7,532	6,465	6843
Sweden	23,689	33,693	25,197	27,556

Change in real net income 2008–2017 in %				
Country	Age 20–39	Age 40–59	Age 60+	Total (20+)
Austria	−1	1	11	3
France	−4	1	8	3
Slovenia	1	4	7	4
Greece	−43	−38	−24	−34
Spain	−18	−8	8	−4
Italy	−17	−9	4	−5
Estonia	17	28	7	17
Poland	5	2	1	2
Sweden	4	16	14	12

The overall pattern of absolute and relative income losses of the young are similar across the whole income distribution. However, mean values can be strongly influenced by large incomes, their changes may not be representative for individuals in the middle or at the bottom end of the distribution. We therefore present changes by quartiles in the Appendix, Tables 5, 6 and 7. While the overall findings hold across the income distribution, we observe a pronounced decline of the first quartile among young adults in Greece, Italy and Spain. This decline can be explained by an increase of the share of persons with a low level of employment and low income to more than one quarter of the population and hence encompasses the first quartile of the income distribution in 2017.

Differences between ESA (Table 1) and EU-SILC data (Table 2) are due to the fact that EU-SILC captures only part of the income of households. These differences are described in detail in Table 8 in the Appendix. In particular, asset income is poorly captured in EU-SILC with the exception of France. Since the decline in income per capita in Austria, Italy and Slovenia (Table 1) was largely the result of a decline in asset income, EU-SILC based income estimates decline less, respectively increased stronger, compared to ESA. Furthermore, changes in the survey may also affect age-specific income. The large increase in income of the working age population in Estonia is partly the result of a better estimation of labour income in EU-SILC; the EU-SILC estimates in 2017 corresponded exactly to the ESA values, while in 2008 the EU-SILC-based estimates corresponded to only 89 percent of the value in ESA. Furthermore, the huge decline in labour income in Greece may be overstated; the estimate of total labour income in EU-SILC was 75 percent of the ESA aggregate in 2017, compared to 89 percent in 2008, suggesting that EU-SILC captured a lower part of total labour income in 2017. By contrast, the estimates of social benefits in EU-SILC increased from 67 to 77 percent of the ESA value, which may explain part of the decline in income of the young relative to the elderly.

The estimation of standard errors is a challenge in any EU-SILC-based analysis. First, the data lacks sample design variables to calculate the correct standard errors due to sampling. Second, further random errors are introduced through imputation and re-weighting of the data (Goedemé 2013), which is particularly problematic for variables with a high share of imputed values, such as asset income. We calculated the standard errors for the age-specific estimates of income changes using the method suggested by Goedemé (2013) and Trindade and Goedemé (2016), and report the results in Table 9 in the Appendix. The standard errors are particularly high in France, which can be explained by the high coverage of unequally distributed asset income and the consequent much higher dispersion of total income. However, a large part of the income data is imputed and not sampled. Treating the data as if it would emerge from a random sample overestimates the standard errors. So far, we are not aware of methodology that enable the estimation of standard errors and confidence intervals of EU-SILC variables with a large share of imputed data. To evaluate our point estimates of income, we carefully compare them with the more reliable aggregate data.

### Age-Specific Changes by Gender

The increase in average income in old age is mainly explained by the substantial increase in female labour force participation in the last decades. As a consequence, women earn more and receive higher pensions than in the past, visible in the strong increase in income of women aged 60+ (Table 3). Although the change was much less pronounced for men, also income of men in the age group 60+ increased, compared to the working age population. Exceptions are Estonia and Poland, where the income of the older male and female population increased less than the income of the working age population.

**Table 3:** Changes in mean net income by age and gender *Source*: Authors’ own calculation based on EU-SILC cross-section data

Country	Men			Women		
	Age	Age	Age	Age	Age	Age
	20–39	40–59	60+	20–39	40–59	60+
Austria	−1	0	6	1	4	16
France	−8	−1	2	2	5	14
Slovenia	3	2	2	−2	7	12
Greece	−41	−41	−28	−44	−34	−19
Spain	−22	−14	4	−12	1	12
Italy	−19	−12	3	−15	−4	5
Estonia	11	29	6	24	26	8
Poland	0	1	−5	10	2	5
Sweden	1	15	12	8	18	17

### Decomposition of Income Changes

**Fig. 1:**
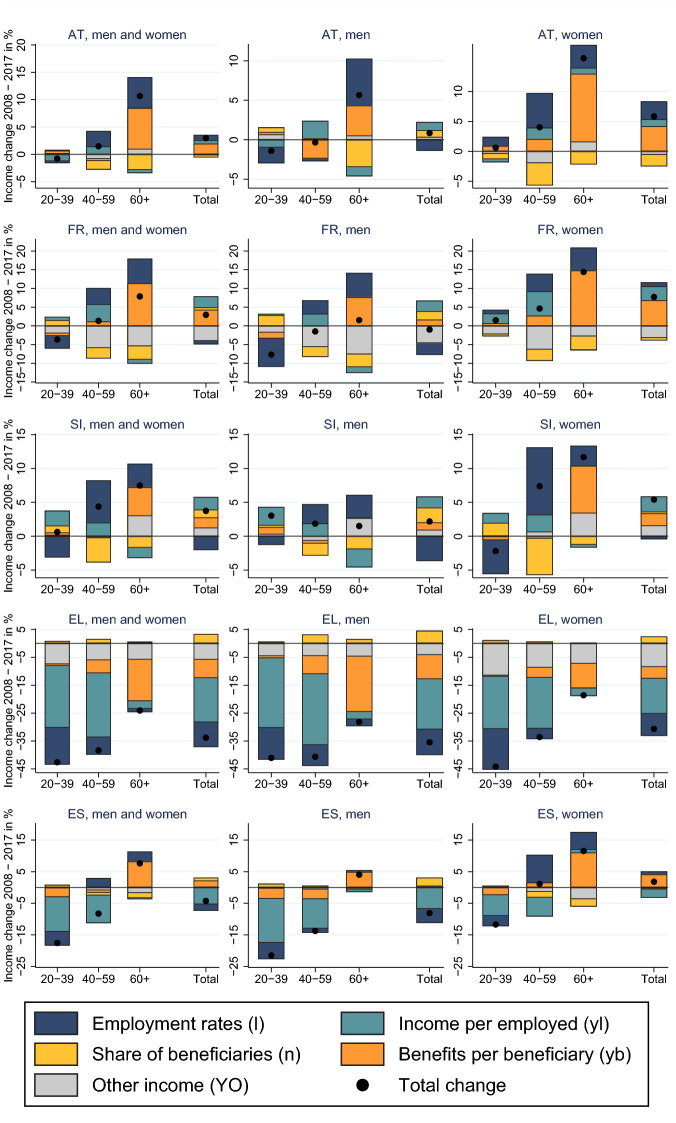

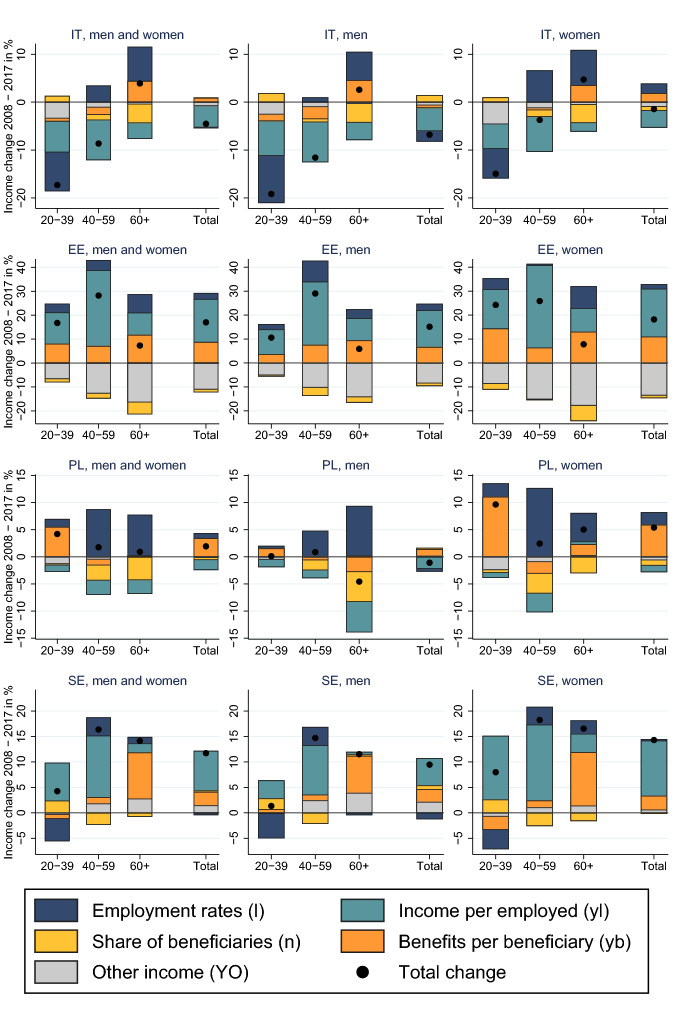
Decomposition of income changes by age

Most of the age-specific differences in income trends can be explained through changing employment rates ($\varDelta _\% l_i$) and an increase in transfer income for the age group 60+ ($\varDelta _\% \text {YB}_{60+}$). Despite huge cross-country differences in the extent of income changes, we observe several common patterns (Fig. 1 and associated Table 4). In all countries, employment among the older working age population and the population 60+ increased, which is mainly the result of higher female employment and later retirement for both genders. Higher employment among older age groups reduced the share of non-employed persons and pension receivers ($\varDelta _\% (1-l_{60+})$) and should have reduced the share of pensions in total income. Instead, in almost all countries, this effect was offset by an increase in benefits per retiree ($\varDelta _\% \text {yb}_{60+}$). Higher employment is the main reason for increasing income of the older working age population aged 40-59; higher employment together with the increase in benefits per retiree is the explanation for the increasing income of the population 60+.

Changes in income per employee ($\varDelta _\% \text {yl}_i$) are an important driver for changes in income of the working age population. The decline in the income of the young working age population in Greece, Italy, and Spain is a combined effect of lower employment rates and lower income per employee. The decline for the older working age population is mainly due to lower income per employee. Likewise, income of the working age population increased in Sweden and Estonia, mainly because of higher income. In Poland, income of the young working age increased because of higher benefits.

In general, the other income components ($\text {YO}_i$) constitute a small part of household income and explain a small part of the income changes with the exception of France and Estonia. In France, this pattern reflects the decline in asset income, an income component which is much better captured in France, compared to other countries. The results for Estonia have to be taken with a grain of salt. The decline in other income is mainly due to a decline in imputed rents in EU-SILC, which we regard unrealistic in its extent and at odds with aggregate data from ESA.

**Table 4:** Decomposition of income changes 2007–2008 (data associated with Fig. 1)

	Total net inc. ($\varDelta _\%$Y)	Employment inc. ($\varDelta _\%$YL)	Public trans. ($\varDelta _\%$YB)	Other inc. ($\varDelta _\%$YO)	Employment ($\varDelta _\%$l)	Inc. per empl. ($\varDelta _\%$yl)	Non-empl. ($\varDelta _\%$(1-l))	Trans. per non-empl. ($\varDelta _\%$yb)
*Austria*								
Age 20–39	−**1**	−2	1	0	0	−1	0	0
Age 40-59	**1**	4	−2	−1	3	1	−2	0
Age 60+	**11**	5	5	1	6	−1	−3	7
**Total (20+)**	**3**	2	1	0	**1**	**1**	−**1**	**2**
France								
Age 20-39	−**4**	−2	1	−2	−3	1	2	−1
Age 40–59	**1**	9	−2	−6	4	5	−3	1
Age 60+	**8**	6	8	−5	7	−1	−4	11
**Total (20+)**	**3**	2	5	−4	−**1**	**3**	**1**	**4**
*Slovenia*								
Age 20–39	**1**	−1	1	0	−3	2	1	0
Age 40–59	**4**	8	−4	0	6	2	−4	0
Age 60+	**7**	2	2	3	3	−2	−2	4
**Total (20+)**	**4**	0	3	1	−**2**	**2**	**1**	**1**
*Greece*								
Age 20–39	−**43**	−35	0	−7	−13	−22	1	−1
Age 40–59	−**38**	−29	−3	−6	−6	−23	2	−5
Age 60+	−**24**	−4	−14	−6	−1	−3	1	−15
**Total (20+)**	−**34**	−25	−3	−6	−**9**	−**16**	**3**	−**7**
*Spain*								
Age 20–39	−**18**	−15	−2	0	−4	−11	1	−3
Age 40–59	−**8**	−6	−2	−1	3	−9	−1	−1
Age 60+	**8**	3	7	−2	3	0	−2	8
**Total (20+)**	−**4**	−7	3	0	−**2**	−**5**	**1**	**2**
*Italy*								
Age 20–39	−**17**	−15	1	−3	−8	−6	1	−1
Age 40–59	−**9**	−5	−3	−1	3	−8	−1	−2
Age 60+	**4**	4	0	0	7	−3	−4	4
**Total (20+)**	−**5**	−5	1	−1	**0**	−**5**	**0**	**1**
*Estonia*								
Age 20–39	**17**	17	7	−7	4	13	−1	8
Age 40–59	**28**	36	5	−13	4	32	−2	7
Age 60+	**7**	17	7	−16	8	9	−5	12
**Total (20+)**	**17**	20	8	−11	**3**	**18**	−**1**	**9**
Poland								
Age 20–39	**4**	0	5	−1	1	−1	0	6
Age 40–59	**2**	6	−4	−1	9	−3	−3	−1
Age 60+	**1**	5	−4	0	7	−3	−4	0
**Total (20+)**	**2**	−1	3	0	**1**	−**2**	**0**	**3**
Sweden								
Age 20–39	**4**	3	2	0	−4	7	2	−1
Age 40–59	**16**	16	−1	2	4	12	−2	1
Age 60+	**14**	3	8	3	1	2	−1	9
**Total (20+)**	**12**	7	3	1	**0**	**8**	**0**	**3**

The bold values indicate total change of income, while non-bold values refer to subcomponents of income

## Discussion

**Fig. 2:**
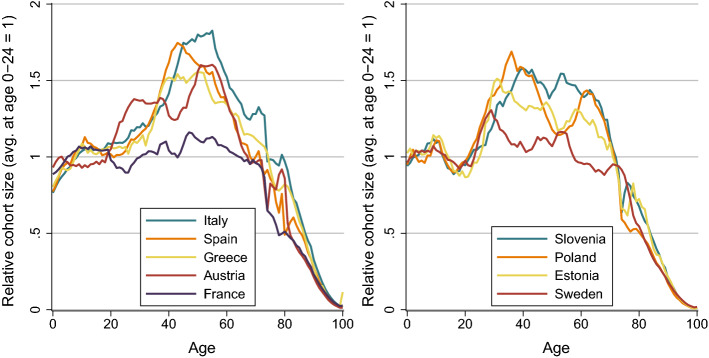
Relative size of birth cohorts in 2020 (avg. size of cohorts aged $0-24 = 1$). *Source*: Eurostat

Understanding age-specific income trends is of central importance in the context of ageing populations and the sustainability of pay-as-you-go pension schemes. Public pension schemes were designed during a period that was characterised by high growth rates of GDP, baby-boom cohorts entering working age, a decline in the number of dependent children, and an increase in the labour force participation of women. These favourable conditions are, however, quickly waning; the size of cohorts entering retirement age in the next years will exceed those entering the labour force by a factor of 1.5, with the exception of France and Sweden (Fig. 2). The change in the age structure of the population will pose a challenge for European economies, in particular the labour markets and public transfer systems. Ageing, its consequences, and possible solutions are therefore widely discussed among policy makers. Only recently, the European commission presented a green paper to launch a broad policy debate on the challenges and opportunities of Europe’s ageing society (European Commission 2021). One of these challenges is the prevention of further income deterioration of young generations. Our results show that the period from 2008 to 2017 was characterised by stagnating/declining income for the young in many countries, increasing taxes, and increasing social benefits for the elderly population. The pressure on income of the young will additionally intensify with the ongoing retirement of the baby-boomer and the consequences of the COVID-19-crisis.

**Fig. 3:**
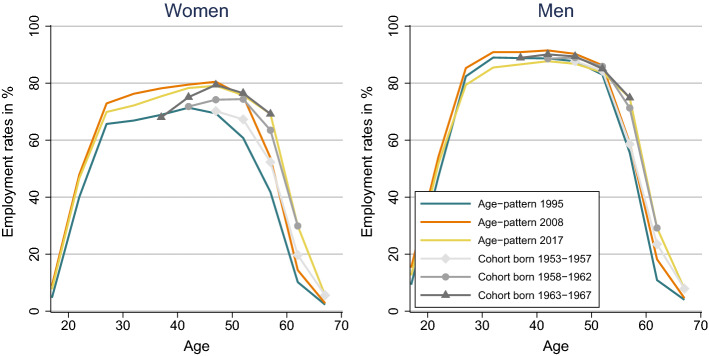
Change in employment by age an birth cohorts, France *Source*: Eurostat

Our results also show that population ageing is only one of the challenges for public transfers in the next decades. The pension systems are also pressured by considerable increases in per-capita benefits. Generous pension rules implemented during demographically favourable periods and the increase in employment resulted in higher pension rights, which explains part of the overall increase in transfer income of the age group 60+. Figure 3 shows the changes in age- and cohort-specific employment rates for the example of France, but the pattern looks very similar in the other countries. In particular, female employment rates have increased in the decades after the baby-boom. Consequently, the cohorts of women that reach retirement age are characterized by higher employment over the whole working life, compared to the already retired cohorts. The increases of female employment rates levelled off for younger age groups after 2008, but increasing employment rates are a major driver of higher labour income in late working age and the increase in public benefits in the age group 60+. The increases in pensions of women are highly desirable, given their low pensions compared to men. They lead, however, to an increase in the average pension compared to income from employment and thereby contribute to the difficulties in balancing contributions and benefits.

While there is no evidence of a straightforward relationship between income and fertility, low income and economic insecurity can explain part of the fertility decline in European countries. The decline in birth rates continued in the years since 2017 and accelerated in the COVID-19 crises, with a particular large drop in Spain (Sobotka et al., 2021). Especially the Southern-European countries may find themselves in a economic vicious circle of demographic and economic developments, where ageing populations result in a shift of income from young to old, which in turn may result in a lower number of children and an acceleration of the ageing process.

The better position of young generations in Poland and Estonia is probably a result of the more dynamic development of the economy in these countries, reflected also in higher growth of GDP per capita. Such developments open work opportunities for younger generations and triggers demand for skills and knowledge that are more widespread among younger persons, e.g. skills regarding information and communications technology, resulting in an increase of their average real wage. An additional factor may be the high net-outmigration in these countries, supporting the wages of the remaining population (e.g. Dustmann et al., 2015; Elsner 2013).

In our sample, all countries with declining national income are characterized by a decrease in income of the young relative to the older population. However, escaping from the vicious circle mentioned above requires institutions that distribute the costs of economic crises across all age groups rather than concentrating the losses of income among the young. A rich literature explores mechanism to adapt pension systems automatically to a changing economic and demographic environment and to balance contributions and expenses (e.g. Börsch-Supan 2007; Vidal-Meliá et al., 2009). Such mechanism could also reduce the differences in age-specific income trends, by avoiding a decoupling of changes in primary income and in pension benefits. In Europe, Sweden is a role model in implementing such stabilizers in the pension system, by adjusting pensions to life expectancy and by linking them to changes in primary income. The constant share of taxes in this country compared to the increase in most other countries suggests that these mechanisms might work as desired (see Table 1). Our work documents age-specific income trends, but further work is needed to identify possible ways towards a sustainable allocation and redistribution of income between generations.

## Conclusion

Our study reveals large differences in age-specific income trends in all nine countries analysed. Although the extent of age-specific differences varies greatly across countries, we observe common patterns.

In most countries, the period 2008–2017 is characterized by a stagnation or decline in the income of households and an increase in public sector redistribution. While GDP per capita decreased only in Greece and Italy between 2008 and 2017, income per capita decreased in six out of the nine analysed countries. Only in Estonia and Sweden, income increased significantly with about 15 percent over the whole period. During the period from 2008 to 2017, income taxes relative to primary income increased in seven of nine countries and stagnated in two, while benefits relative to primary income increased in all countries.

The age group 20–39 lost income relative to older age groups in all countries except Estonia and Poland. In five out of nine countries, the young lost even in absolute terms. The differences in age-specific income trends are particularly high in Southern Europe. In Italy and Spain, mean income in the population at age 20–39 declined by about 17 percent and for the older working age population at age 40–59 by about eight percent. By contrast, income increased for the elderly population aged 60+. In Greece, income declined for all age groups, but less for the older populations. In Austria, France, Slovenia and Sweden, mean income of the population 20–39 merely stagnated, while it increased strongly for the population aged 60 and older.

A decomposition analysis revealed that the main drivers of these age-specific differences in income trends are (i) a decline or stagnation of employment rates and income of the 20–39-year-old, (ii) an increase in employment in the older age groups 40–59 and 60+, and (iii) a strong increase in benefits for the population 60+. The increase in employment and income among older population is mainly due to a increased labour force participation and higher pensions for women.

In summary, this paper revealed important intergenerational disparities in the development of individual income, especially in countries that were hit hard during the previous financial crisis. These findings are crucial with regard to the current COVID-19 pandemic, with its unprecedented societal and economic consequences. For many young Europeans, the pandemic adds to their already precarious economic situation. Knowledge about age-specific income trends may help to find better and generationally balanced answers to economic crises.

### Electronic supplementary material

Below is the link to the electronic supplementary material.

Supplementary material 1 (XLSX 82 kb)

Supplementary material 2 (PDF 274 kb)

## Data Availability

The tables included in the paper are also provided as supplementary material in Excel format.

## Appendix

### Allocation of Household-Level Income to Individuals

Some income components in EU-SILC are only given at household level and need to be assigned to individuals for this analysis. Family benefits are assigned to the parents of the economically dependent children in the household. Since parental leave benefits as part of total family benefits are targeted at the person taking over most of the care responsibilities, we distribute family benefits within couples according to the inverse share of their labour income. If one of the partners has no income because of the engagement in care work, this partner is assumed to receive all family benefits.

Imputed rent is regarded as a type of asset income and assigned to the household members that report to be responsible for the accommodation. Asset income is assumed to be equally shared among all adults in the household.^2^ Finally, personal income of persons below 16 is assigned to the 15-year-old members, or the oldest child in case there are no 15-year-old present in the household.

We want to emphasize again that these allocation rules have no strong effect on our results. An alternative allocation of household-level income to all adults in equal shares resulted in negligible differences in the total changes of income.

### Decomposition of Income Changes

This section describes the decomposition of the changes in average income from employment $\varDelta _\% \mathrm {YL}_{i}$ and average benefits $\varDelta _\% \mathrm {YB}_{i}$ for each age group i, and provides an illustrative example at the end of the section.

The decomposition relies on information about employment during the income reference period (the calendar year preceding the year of the survey). To calculate employment rates, we use the information on economic status in each month (Variables PL073-PL076 in EU-SILC). For example, an observation counts as fully employed if he or she reports employment for 12 months. If an observation, however, reports only six months in employment, he or she is considered as half employed and half non-employed. We refrain from further accounting for the extent of employment, since we do not have information on the exact numbers of hours worked. Note, that also individuals who are fully employed may receive public transfers, e.g. child allowance. Most of public transfers, however, target individuals outside employment via pensions, parental leave benefits or unemployment benefits. These are also the components that account for the income changes over time.

Remember that in our analysis we measure the change of each income component relative to total income $Y_i$. For example, $\varDelta _\% \mathrm {YL}_i=\frac{\mathrm {YL}_{i,2017} - \mathrm {YL}_{i,2008}}{\mathrm {Y}_{i,2008}}$. We can also write $\varDelta _\% \mathrm {YL}_i$ as percentage change of $\mathrm {YL}_i$ (indicated by $\delta _\% \mathrm {YL}_i$), scaled with the share of $\mathrm {YL}_i$ in total income: $\frac{\mathrm {YL}_{i,2017} - \mathrm {YL}_{i,2008}}{\mathrm {YL}_{i,2008}}*\frac{\mathrm {YL}_{i,2008}}{\mathrm {Y}_{i,2008}} = \delta _\% \mathrm {YL}_i*\frac{\mathrm {YL}_{i,2008}}{\mathrm {Y}_{i,2008}}$. Likewise, $\varDelta _\% \mathrm {YB}_i$ is the percentage change of $\mathrm {YB}_i$ scaled with its share in total income: $\varDelta _\% \mathrm {YB}_i = \delta _\% \mathrm {YB}_i*\frac{\mathrm {YB}_{i,2008}}{\mathrm {Y}_{i,2008}}$.

We write $\mathrm {YL}_i$ as product of employment rate ($l_i$) and average income of each employed person ($\text {yl}_i$): $\text {YL}_i = \text {yl}_i*l_i$. Because public benefits are mainly directed at persons not in employment, such as pensioners, unemployed persons or parents on leave, we can write average benefits as product of the share of non-employed persons $(1-l_i)$ and average benefits per non-employed person ($\text {yb}_i$): $\text {YB}_i = \text {yb}_i*(1-l_i)$. Because the decomposition-approach is the same for $\text {YL}_i$ and $\text {YB}_i$, we illustrate it now only for income from employment $\text {YL}_i$.

In continuous time, i.e. if we would look at an infinitesimal time interval, we could calculate the percentage change of $\text {YL}_i$ as derivative of the logarithm with respect to time and write it as sum of the percentage change of each of the two factors: $\frac{d}{{dt}}\ln \mathrm {YL(t)}_i = \frac{d}{{dt}}\ln \left( l(t)_i*\text {yl(t)}_i\right) =\frac{d}{{dt}}\ln l(t)_i + \frac{d}{{dt}}\ln \text {yl(t)}_i$.

Such an approach is not completely correct for our discrete, ten-year interval. The percentage changes $\delta _\% \text {yl}_i$ and $\delta _\% \text {l}_i$ do not exactly add up to $\delta _\% \text {YL}_i$. However, even for our 10-year time interval the difference is very small. Therefore, we approximate the additive contributions of each factor to $\delta _\% \text {YL}_i$ by their percentage change. To ensure that the components add up, we calculate $\delta _\% \text {yl}_i$ as residual: $\delta _\% \text {yl}_i = \delta _\% \text {YL}_i - \delta _\% l_i$. To measure the contributions of each of these factors in terms of total income, we rescale $\delta _\% \mathrm {yl}_i$ and $\delta _\% \mathrm {l}_i$ by $\frac{\text {YL}_{i,2008}}{\text {Y}_{i,2008}}$. Using this procedure also for the benefits, we can write the total changes in income as sum of the five components (Equation 4 in Sect. 2.3):

5 $$\begin{aligned} \varDelta _\% \text {Y}_i = \varDelta _\% \mathrm {l}_{i} + \varDelta _\% \mathrm {yl}_{i} + \varDelta _\% (1-\text {l}_{i}) + \varDelta _\% \text {yb}_{i} + \varDelta _\% \text {YO}_{i} \end{aligned}$$

For example, in Spain the change in average employment-income for all adults aged 20+ (the group ”Total” in Table 4) $\delta _\% \text {YL}_{20+}$ was -13 percent. The employment rate decreased by four percent ($\varDelta l_{20+} = -4$). We approximated the change in income per employed person: $\delta \text {yl}_{20+} = \delta \text {YL}_{20+} - \delta l_{20+} = -13 - (-4) = -9$. Because income from employment ($\text {YL}_{20+}$) amounted to 57 percent of total income ($\text {Y}_{20+}$), we rescaled these numbers to measure their change in terms of total income: $\varDelta _\% \mathrm {YL}_{20+} = 9*0.57 \approx \mathrm {5~percent}$ and $\varDelta _\% \mathrm {l}_{20+} = 4*0.57 \approx \mathrm {2~percent}$. The two components together add up to the change of -7 percent in employment income $\varDelta _\% \mathrm {YL}_{20+}$.

### Age-Specific Income Changes: Percentiles

See Table 5, 6 and 7

**Table 5:** Net income and its changes 2008 - 2017, $1^{st}$ Quartile *Source*: Eurostat, EU-SILC

Change in real net income 2008–2017 in %				
Country	Age 20-39	Age 40-59	Age 60+	Total
Austria	2	4	11	6
France	−8	4	10	3
Slovenia	−5	4	9	5
Greece	−89	−43	−9	−39
Spain	−49	−2	14	− 13
Italy	−47	−10	3	−16
Estonia	13	21	−1	3
Poland	36	9	3	15
Sweden	1	15	12	9

**Table 6:** Median net income and its changes 2008 - 2017 *Source*: Eurostat, EU-SILC

Change in real net income 2008–2017 in %				
Country	Age 20–39	Age 40–59	Age 60+	Total
Austria	3	2	10	4
France	− 2	1	10	3
Slovenia	2	6	6	4
Greece	− 50	− 33	− 16	− 28
Spain	− 24	− 7	9	− 4
Italy	−26	−7	7	−3
Estonia	23	28	−6	14
Poland	16	10	3	9
Sweden	6	18	12	12

**Table 7:** Net income and its changes 2008 - 2017, $3^{rd}$ Quartile *Source*: Eurostat, EU-SILC

Change in real net income 2008–2017 in %				
Country	Age 20–39	Age 40–59	Age 60+	Total
Austria	0	−1	10	2
France	−1	2	8	2
Slovenia	1	5	5	4
Greece	−38	−38	−22	−31
Spain	−11	−8	9	−1
Italy	−11	−8	6	−3
Estonia	20	32	6	24
Poland	6	1	2	2
Sweden	6	18	17	15

### Explaining the Difference between Aggregate Income in ESA and EU-SILC

Table 8 compares EU-SILC and ESA data by measuring the share of aggregate income that is covered in EU-SILC by type of income (coverage ratio, CR). Furthermore, it shows the change of the CR between 2008 and 2017. In general, wages and social benefits are similar in both surveys. Differences between ESA and EU-SILC are mainly due to large differences observed for asset income (Törmälehto, 2019). The CR for total income increased considerably in Austria, Italy and France. In Austria and Italy, this increase can be explained with the decline in asset income. Since only a small part of asset income is captured in EU-SILC, the decline in this component increases the share of total income that is captured. In both countries, the CR of employment income and social benefits actually decreased. In France, where asset income may be over-covered by EU-SILC (Törmälehto, 2019), the increase in the total CR is explained by an increase in the CR of both employment income and social benefits. In Estonia, the coverage of income from employment and of social benefits increased, but not the coverage of total income. We thus observe a unrealistic, strong decline of imputed rents in EU-SILC, reducing the coverage of total income. In Greece, the CR of employment income decreased strongly (-14 per cent), while the CR of social benefits increased by 10 per cent.

**Table 8:** The coverage ratio of EU-SILC over time: aggregate income in ESA compared to EU-SILC

Country	2017 coverage ratio (CR)			Change CR 2008–2017		
	Total income	Employment income	Social benefits	Total income	Employment income	Social benefits
Austria	86	98	83	5	−1	0
France	99	91	86	5	4	5
Slovenia	86	82	87	3	−2	0
Greece	74	75	77	1	−14	10
Spain	88	89	84	1	1	5
Italy	86	97	87	6	−3	−2
Estonia	82	101	72	0	12	10
Poland	74	91	75	−1	2	0
Sweden	85	110	83	−4	−1	−6

### Standard Errors of Income Changes

See Table 9 .

**Table 9:** Standard errors of income changes

Standard errors of the estimated income changes 2008–2017				
Country	Age 20–39	Age 40–59	Age 60+	Total
Austria	2.2	2.8	2.0	1.4
France	4.2	6.8	10.0	6.4
Slovenia	1.6	1.6	1.6	0.9
Greece	4.2	6.4	4.2	3.5
Spain	2.5	3.2	2.4	1.9
Italy	3.1	5.4	4.6	4.1
Estonia	2.7	2.0	1.3	1.3
Poland	2.2	1.9	2.2	1.5
Sweden	1.6	2.8	2.4	1.4

## Footnotes

1 For example, $$\varDelta _\% \mathrm {YL}_i=\frac{\mathrm {YL}_{i,2017} - \mathrm {YL}_{i,2008}}{\mathrm {Y}_{i,2008}}$$.

## Code Availability

The code for the age-specific analysis and the decomposition is provided as supplementary material to the paper. It requires access to EU-SILC micro-data.
