# Supplementary material for: Age-Specific Income Trends in Europe: The Role of Employment, Wages, and Social Transfers
Source: Soc Indic Res. 2021 Nov 30;162(2):525–47. doi: 10.1007/s11205-021-02838-w (PMC8629699; doi:10.1007/s11205-021-02838-w)
Supplement: Supplementary file 2 — Supplementary material 2 (PDF 274 kb) [file 11205_2021_2838_MOESM2_ESM.pdf]

```
* Decomposition of income changes

* The code produces descriptive data and the decomposition of age-specific income
changes as published in:

* Article Title : Age-Specific Income Trends in Europe: The Role of Employment,
Wages, and Social Transfers
* Journal: Social Indicators Research
* Authors: Bernhard Hammer (bernhard.hammer@tuwien.ac.at), Sonja Spitzer, Alexia
Prskawetz
* DOI : 10.1007/s11205-021-02838-w

* Input: Merged EU-SILC cross-section data

********************************************************************************
* Content
* 0. Merge the data delivered by Eurostat and transform it to STATA format
* 1. Generate needed variables
  * 1.1 Needed non-income variables
  * 1.2 Needed income variables
* 2. Descriptive statistics: age-specific income means & change
* 3. Descriptive statistics: age-specific income percentiles & change
* 4. Means and percentiles: Copy and paste to Excel file; Table 2,3,5,6 and 7
* 5. Decomposition of age-specific income changes: Table 4
* 6. Graphs of age-specific income changes, Fig. 1
********************************************************************************

* Working directory
  local wdir "C:\Users\bhammer\EUSILC\data"
  cd `wdir'

* Folder with the original SILC data
  global data_original "D:\EUSILCdata\Cross\"

* Folder for graphs
  global graphslocation "C:\Users\bhammer\EUSILC\graphs"


global country AT IT FR SE SI PL EE EL ES
global year 04 05 06 07 08 09 10 11 12 13 14 15 16 17 18 19



**********************************************************************************
*************************************************
* 0. Merge the data delivered by Eurostat and transform it to STATA format
**********************************************************************************
*************************************************

foreach x in $country {
```

```
foreach y in $year {

  ******************************************************************************
  * 0.1 Read csv files, convert the into stata files
  ******************************************************************************


  * Household register file
    clear all
    cap noi insheet using "$data_original\`x'\20`y'\UDB_c`x'`y'D.csv", names case
delim(",")
        cap noi clonevar HHID = DB030
        gen YEAR = 1999 + `y' // YEAR refers to income reference period
        cap noi sort HHID
    cap noi save "UDB_c`x'`y'D", replace

  * Personal register
    clear all
    cap noi insheet using "$data_original\`x'\20`y'\UDB_c`x'`y'R.csv", names case
delim(",")
    cap noi clonevar PID = RB030    // personal ID
    cap noi gen HHID = RX030
    cap noi sort HHID PID
    cap noi save "UDB_c`x'`y'R", replace

  * Household data
    clear all
    cap noi insheet using "$data_original\`x'\20`y'\UDB_c`x'`y'H.csv", names case
delim(",")
    cap noi save "UDB_c`x'`y'H", replace
    cap noi clonevar HHID = HB030
        cap noi sort HHID
    cap noi save "UDB_c`x'`y'H", replace

  * Personal data
    clear all
    cap noi insheet using "$data_original\`x'\20`y'\UDB_c`x'`y'P.csv", names case
delim(",")
        cap noi destring PB120, ignore ("**")replace // in Poland there is **
instead of number
    cap noi clonevar PID = PB030
        cap noi gen HHID = PX030
        * Alternative if PX030 missing
      cap noi gen HHID_1 = PID/100   // last two numbers is the personal identifyer
within the household
      cap noi gen HHID1 = floor(HHID_1)
      cap noi replace HHID = HHID1 if HHID==.
    cap noi sort HHID PID
    cap noi save "UDB_c`x'`y'P", replace
        display "`x'"
```

```
        display "`y'"

    ************************************************************
    * 0.2. Merge the files for a given year and a given country
    ************************************************************

    * Start with register data
      clear all
      cap noi use "UDB_c`x'`y'R", clear
      cap noi sort HHID PID

    * merge personal data
      cap noi merge HHID PID using UDB_c`x'`y'P.dta
      cap noi tab _merge  // children below 16 are only in register data
      cap drop _merge

    * merge household data
      cap noi sort HHID
      cap noi merge m:1 HHID using UDB_c`x'`y'H.dta
      cap noi tab _merge
      cap drop _merge

    * merge household register data
      cap noi sort HHID
      cap noi merge m:1 HHID using UDB_c`x'`y'D.dta
      cap noi tab _merge
      cap drop _merge

    * Save data by country and year
      cap noi sort HHID PID
      cap noi save silc`x'`y', replace
          display "`x'"
          display "`y'"

    * Erase the single data files
      cap noi erase UDB_c`x'`y'H.dta
          cap noi erase UDB_c`x'`y'P.dta
          cap noi erase UDB_c`x'`y'R.dta
          cap noi erase UDB_c`x'`y'D.dta

  }


    ******************************************************************************
    * 0.3Cross-section data; create dataset with all years for each country
    ******************************************************************************

    local firstyear = "0"

    * Append the yearly data for each country
```

```
        foreach y of global year {
            capture confirm file silc`x'`y'.dta // is there a data set for the year
and country
                if (_rc==0 & `firstyear'==0) {      // if it is the first year in
the survey, load the data
                use silc`x'`y'.dta, clear
                    *erase silc`x'`y'.dta
                    local firstyear = `y'           // generate a local indicating
the first year
                }
            if (_rc==0 & `firstyear'!=`y') {    // append the later years
                append using silc`x'`y'.dta, force
                }
            if (_rc==0) {
                  erase silc`x'`y'.dta
                }
            }
    save silc`x'0_1, replace
        display "`x'"
        display "`y'"

}



************************************************************************************
*************************************************
* 1. Generate needed variables
************************************************************************************
*************************************************

foreach x in $country {
  use silc`x'0_1, clear

********************************************************************************
* 1. Needed non-income variables
********************************************************************************

  * Country
      gen CNT2 = "`x'"

 * Age at end of income reference period
      gen AGE = RX020
      label var AGE "Age at end of inc reference period"

 * Age groups
        gen AGEGR20 = 0 if AGE<20
            replace AGEGR20 = 2 if AGE>=20 & AGE<40
            replace AGEGR20 = 4 if AGE>=40 & AGE<60
            replace AGEGR20 = 6 if AGE>=60
```

```
          label define AGEGR20 0 "<20" 2 "20-39" 4 "40-59" 6 ">60"
          label val AGEGR20 AGEGR20

   * Sex
     gen SEX = RB090
          label define SEX 0 "Total" 1 "male" 2 "female"
          label val SEX SEX


  * Personal weight
       gen WEIGHT = RB050 // personal cross-sectional weight

   * Household size
          bysort YEAR HHID: egen HHSIZE = count(PID)
            label var HHSIZE "no. of hh members"

   * Within household ID
       // Ranging from 1 to number of household members
       // Within household ID is used to avoid problems with the large numbers of the
personal ID
       gen hhid = PID/100   // last two numbers is the personal identifyer within the
household
       replace hhid = floor(hhid) // cut the hid off the pid
       gen PIDHH = PID - hhid*100 // number within household
           label var PIDHH "ID of HH-member within household"
           drop hhid


   ***************************
   * Identify adult persons: those most likely to own assets and generate income
   ***************************

       * If person below 35, he/she must be in employment (PL031: 1-4) or doing
domestic work (PL031: 10)
         gen ADULT = 0
            replace ADULT = 1 if (((PL031>=1 & PL031<=4) | PL031==10) & AGE>=16) |
AGE>=35

           * If there is not working adult, also younger non-working persons are
treated as adult
            bysort YEAR HHID: egen NADULT = total(ADULT)
            replace ADULT = 1 if AGE>=16 & NADULT==0

            drop NADULT
            bysort YEAR HHID: egen NADULT = total(ADULT)

          // In Italy or Austria there are no households with only persons below 16

            replace ADULT = 1 if AGE>14 & NADULT==0
```

```
          * Households without adult persons (15+) are droped
            drop NADULT
            bysort YEAR HHID: egen NADULT = total(ADULT)
            drop if NADULT==0


  *****************
  * Activity status
  *****************
    gen ACTIVITY = .

        * Value labels for activity variable
      label define ACTIVITY 0 "missing" 1 "education" 2 "parttime" 3 "fulltime" ///
                            4 "unemployed" 5 "domestic work and care" 6 "retired"
///
                            7 "inactive, other"
          label values ACTIVITY ACTIVITY

        * Use information from PL031, self-defined economic status after 2008
      replace ACTIVITY = 1 if PL031==6 | PL031==9 | AGE<=15 // education and unpaid
work experience, military service
      replace ACTIVITY = 2 if PL031==2 | PL031==4 // employed and self-employed part
time
      replace ACTIVITY = 3 if PL031==1 | PL031==3 // employed and self-employed
fulltime; compulsory military and civil service
      replace ACTIVITY = 4 if PL031==5 // unemployed
      replace ACTIVITY = 5 if PL031==10 // domestic work
      replace ACTIVITY = 6 if PL031==7 | PL031==8  // retired or disabled
      replace ACTIVITY = 7 if PL031==11 // inactive other

        * Use information from PL030, self-defined economic status before 2009
      capture replace ACTIVITY = 1 if PL030==4 | PL030==7 | AGE<=15 // education and
unpaid work experience, military service
      capture replace ACTIVITY = 2 if PL030==2 // working part time
      capture replace ACTIVITY = 3 if PL030==1  // working fulltime; compulsory
military and civil service
      capture replace ACTIVITY = 4 if PL030==3 // unemployed
      capture replace ACTIVITY = 5 if PL030==8 // domestic work
      capture replace ACTIVITY = 6 if PL030==5 | PL030==6  // retired or disabled
      capture replace ACTIVITY = 7 if PL030==9 // inactive other

        * Complement info and replace missing values using other variables
        * (RB170 main activity status during income reference period)
      replace ACTIVITY = 1 if PE010==1 & ACTIVITY==. // current education activity:
in education
      replace ACTIVITY = 3 if RB210==1 & ACTIVITY==. // at work (no info about
full-time and part-time)
      replace ACTIVITY = 4 if RB210==2 & ACTIVITY==.  // unemployed
      replace ACTIVITY = 6 if RB210==3 & ACTIVITY==.  // retired
      replace ACTIVITY = 7 if RB210==4 & ACTIVITY==.  // inactive, other
```

```
      replace ACTIVITY = 6 if ACTIVITY==. & AGE>=60  // retired if age>60 and
missing
          replace ACTIVITY = 6 if AGE>=60 & ACTIVITY!=2 & ACTIVITY!=3

          replace ACTIVITY = 3 if ACTIVITY==. & (PY010G>=8000 | PY050G>=8000)


  ***********************
  * Person responsible for accomodation
  ***********************

      gen ACCOMODATION = 0
      replace ACCOMODATION = 1 if HB080==PID // Person 1
      replace ACCOMODATION = 1 if HB090==PID // Person 2 responsible for
accomodation
          label var ACCOMODATION "Persons responsible for accomodation"

          * In each household there has to be at least one person responsible for
accomodation
            bysort YEAR HHID: egen NACCOMOD = total(ACCOMODATION)
            egen ACCOTAG = tag(YEAR HHID ADULT)
            replace ACCOMODATION = 1 if ACCOTAG==1 & ADULT==1 & NACCOMOD==0
        drop NACCOMOD ACCOTAG

 *****************************
  * Characteristics of partner
  *****************************

    preserve

    drop if RB240==.   // drop if partner is not living in the same household

    * Choose partner-characteristics of interest
      local partnerchar "AGE ACTIVITY ACCOMODATION PIDHH"
            keep `partnerchar' RB030 RB240 YEAR
        rename RB240 PID // for merging with main data partner-ID is ID, and ID
becomes partner-ID
            rename RB030 PID_P

    * Partner-variables are are named as [variable]_P
      foreach var of local partnerchar {
            rename `var' `var'_P
          }

    * Save the file with partner-data
          sort YEAR PID
      tempfile partnerdata
          save `partnerdata'

    restore
```

```
    * Merge full dataset with the partner dataset
          sort YEAR PID
          merge YEAR PID using `partnerdata'
          drop _merge


  *****************************
  * Couple ID: Identify and number persons living within couples within HH
  *****************************

  // Numbering of couples within household:
  // Each couple within the household gets a number ranging from 1
  // to the number of couples in the HH

  gen COUPLEID = .
    label var COUPLEID "Couple ID within HH"

  sum HHSIZE
        global hhsizemax `r(max)'

  * Assign to each couple the personal ID with the lowest value (couple PIDHH==2 and
PIDHH==4 gets COUPLEID = 2)
    forvalues i = 1/$hhsizemax {
          replace COUPLEID = `i' if (PIDHH==`i' | PIDHH_P==`i') & COUPLEID==.
          replace COUPLEID = . if PIDHH_P==.
    }

  * Ensure that COUPLEID is a sequence without holes
          egen COUPLETAG = tag(YEAR HHID COUPLEID)
          bysort YEAR HHID: egen COUPLEIDSEQ = seq() if COUPLETAG==1
          rename COUPLEID COUPLEIDtemp
          bysort YEAR HHID COUPLEIDtemp: egen COUPLEID = total(COUPLEIDSEQ)
          replace COUPLEID = . if COUPLEIDtemp==.
          drop COUPLEIDtemp COUPLEIDSEQ

  ***************************************************************
  * Survey design: strata and primary sampling units
  ***************************************************************

    * Generates the variables with the primary sampling unit and strata
      * Approach:
      * PSUs are identified by DB060
      * DB040 (NUTS1/NUTS2 regions) are used as a proxy for for the stratum variable

      * DB040 underestimates the number of strata in BE CZ GR ES FR IT RO

      * Italy has self-representing PSUs: In this case there should be only one PSU
within the Stratum
```

```
    browse YEAR HHID DB040 DB060

* Number of observations
  bysort YEAR: egen NOBS = count(PID)

* Identifier of STRATA
  capture confirm numeric variable DB040
  if !_rc {
    gen DB040miss1 = 1 if DB040==.
  }
  else {
      gen DB040miss1 = 1 if DB040==""
    }
    bysort YEAR: egen DB040miss = total(DB040miss1)
    drop DB040miss1
  *egen DB040miss = count(DB040)
    if DB040miss==0 {
    encode DB040, gen(DB040n)
      gen STRATA = DB040n
    }
    else {
      gen STRATA = 1
    }
  * If DB040 is missing we assume a single stratum
  // cap noi egen STRATAMISS = count(DB040n)
  // cap noi replace STRATA = 1 if STRATAMISS==0
  // cap noi drop STRATAMISS

* Generate one Stratum for those with missing Strata
  sum STRATA
    replace STRATA = `r(sum)'+1 if STRATA==.

* Identifier of PSUs
  gen PSU = DB060

* Number of PSUs
  sort YEAR PSU
  gen PSU1 = 1 if (PSU[_n]!=PSU[_n-1]) & PSU!=.
  bysort YEAR: egen NPSU = sum(PSU1)
  drop PSU1

* Number of observ. with missing PSU
  bysort YEAR: egen NPSUMISS = total(PSU==.)

* Number of strata
  sort YEAR STRATA
  gen STRATA1 = 1 if (STRATA[_n]!=STRATA[_n-1]) & STRATA!=.
  bysort YEAR: egen NSTRATA = sum(STRATA1)
  drop STRATA1
```

```
  * Number of obsrvations with missing strata
    bysort YEAR: egen NSTRATAMISS = total(STRATA==.)

  * Strata not unique within PSUs
    bysort YEAR PSU: egen STRATAmin = min(STRATA)
    bysort YEAR PSU: egen STRATAmax = max(STRATA)
        gen STRATA_PSU1 = 1 if STRATAmin!=STRATAmax
        bysort YEAR: egen STRATA_PSU = sum(STRATA_PSU1)
        drop STRATA_PSU1

  * PSU not unique within HHID
    bysort YEAR DB030: egen PSUmin = min(PSU)
    bysort YEAR DB030: egen PSUmax = max(PSU)
        gen PSU_HHID1 = 1 if PSUmin!=PSUmax
        bysort YEAR: egen PSU_HHID = sum(PSU_HHID1)
        drop PSU_HHID1

    egen YEARTAG = tag(YEAR)

        drop NPSU NPSUMISS NSTRATA NSTRATAMISS PSUmin PSUmax STRATAmin STRATAmax
STRATA_PSU PSU_HHID

  * If DB060 is missing or a large share of them we take households as PSUs
          bysort YEAR: egen PSUMISS = sum(PSU==.)
          gen PSUMISSsh = PSUMISS/NOBS
        replace PSU = DB030 if PSUMISSsh>0.5
        replace PSU = DB030 if PSU==.
          drop PSUMISS PSUMISSsh

  * Replace missing PSU with household ID
    replace PSU = HHID if PSU==.

  * Assign Split-PSUs to one Stratum
    bysort YEAR PSU: egen MEDSTRAT = mode(STRATA)
        replace STRATA = MEDSTRAT if STRATA!=MEDSTRAT

  * Number of PSUs
    sort YEAR PSU
    gen PSU1 = 1 if (PSU[_n]!=PSU[_n-1]) & PSU!=.
    bysort YEAR: egen NPSU = sum(PSU1)
    drop PSU1

  * Number of observ. with missing PSU
    bysort YEAR: egen NPSUMISS = total(PSU==.)

  * Number of strata
    sort YEAR STRATA
    gen STRATA1 = 1 if (STRATA[_n]!=STRATA[_n-1]) & STRATA!=.
    bysort YEAR: egen NSTRATA = sum(STRATA1)
    drop STRATA1
```

```
  * Number of obsrvations with missing strata
    bysort YEAR: egen NSTRATAMISS = total(STRATA==.)

  * Strata not unique within PSUs
    bysort YEAR PSU: egen STRATAmin = min(STRATA)
    bysort YEAR PSU: egen STRATAmax = max(STRATA)
        gen STRATA_PSU1 = 1 if STRATAmin!=STRATAmax
        bysort YEAR: egen STRATA_PSU = sum(STRATA_PSU1)
        drop STRATA_PSU1

  * PSU not unique within HHID
    bysort YEAR DB030: egen PSUmin = min(PSU)
    bysort YEAR DB030: egen PSUmax = max(PSU)
        gen PSU_HHID1 = 1 if PSUmin!=PSUmax
        bysort YEAR: egen PSU_HHID = sum(PSU_HHID1)
        drop PSU_HHID1



********************************************************************************
* 1.2 Needed income variables
********************************************************************************

  ************************
  * Replace missing values
  ************************

    * Labour income and asset income (primary income)

      local income "PY010G PY020G PY021G PY030G PY050G PY010N PY020N PY021N PY050N"
      // PY010 employee cash or near cash income
          // PY020 non-cash employee income; PY021 company car
          // PY030 employers' social contributions (only gross)
          // PY050 income and losses from self-employment
          foreach var of local income {
              replace `var' = 0 if `var'==.
          }

          gen PYPG = PY010G + PY020G + PY050G
            label var PYPG "Personal primary income gross"

          gen PYPN = PY010N + PY020N + PY050N
            label var PYPN "Personal primary income net"

    * Public transfers

      local income "PY090G PY100G PY110G PY120G PY130G PY140G PY090N PY100N PY110N
PY120N PY130N PY140N"
          foreach var of local income {
```

```
            replace `var' = 0 if `var'==.
        }
    // PY090 Unemployment benefits
        // PY100 Old age benefits
        // PY110 Survivor benefits
        // PY120 Sickness benefits
        // PY130 Disability benefits
        // PY140 Education related allowances

  * Other income components

    local income "PY035G PY080G PY035N PY080N"
        foreach var of local income {
            replace `var' = 0 if `var'==.
        }
    // PY035 contributions to private pension plan (net values avilable only for
some years)
    // PY080 income from private pension plan (net values available only for some
years)

  * Primary income

    local income "HY030G HY040G HY090G HY110G HY170G HY030N HY040N HY090N HY110N
HY170N"
        foreach var of local income {
          replace `var' = 0 if `var'==.
        }
        // HY030 Imputed rent
        // HY040 Income from rental of property or land
        // HY090 Income from capital investments
        // HY110 Income received by persons below 16
        // HY170G Value of goods produced for own consumption

        gen HYPG = HY040G + HY090G + HY110G
          label var HYPG "HH primary income gross"

        gen HYPN = HY040N + HY090N + HY110N
          label var HYPN "HH primary income net"

  * Transfers at household level

    local income "HY050G HY060G HY070G HY080G HY050N HY060N HY070N HY080N"
        foreach var of local income {
          replace `var' = 0 if `var'==.
        }
        // HY050 Family and child allowances
        // HY060 Social exclusion n.e.c.
        // HY070 Housing allowances
        // HY080 Inter-household cash transfer received
```

```
          gen HYTG = HY050G + HY060G + HY070G + HY080G
            label var HYTG "HH transfers gross"

          gen HYTN = HY050N + HY060N + HY070N + HY080N
            label var HYTN "HH transfers net"

    * Expenditures

      local expenditure "HY100G HY120G HY130G HY140G HY100N HY120N HY130N HY140N
HY145N"
          foreach var of local expenditure {
            replace `var' = 0 if `var'==.
          }
          // HY100 Interest repayment on mortgage
          // HY120 Regular taxes on wealth
          // HY130 inter household cash transfer paid
          // HY140 Tax on income and social contributions
          // HY145 Repayments/receipts from tax adjustments (only net)

    * Disposable income
      replace HY020 = 0 if HY020==.


  *****************************
  * Individualize family and child benefits
    * (goes to parents of children in education)
  ****************************

    gen PYG = PY010G + PY020G + PY050G + PY080G + PY090G + PY100G + PY110G + PY120G
+ PY130G + PY140G

    tempfile data
    save `data', replace

    * Information on the children of fathers (RB220): number and education status
****************
          // generate the desired information for children, save data, and merge it
with the data of the father

          * Education status
            gen EDUC = 0
              replace EDUC = 1 if ACTIVITY==1 & AGE<35
              label var EDUC "in education and younger than 35"

          * Keep the information for children whose father is identified in the
survey
            keep EDUC RB220 HHID YEAR
            drop if RB220==.  // drop if father is not living in the same household
        rename RB220 PID // father ID becomes ID - for merging
```

```
      * Identify siblings (children of a certain person)
        egen SIBLING = group(YEAR HHID PID)  // group all children of a person who
live in the HH
        sort SIBLING

      * Number of children in education
            by SIBLING: egen EDUCHILD=total(EDUC==1)
              label var EDUCHILD "number of children in education"

      * Number of children
            bysort YEAR HHID SIBLING: egen NCHILD=count(PID)
              label var NCHILD "number of children"

          * Select "representative" sibling
        egen SIBLINGTAG = tag(SIBLING)
        keep if SIBLINGTAG==1

          * Save child dataset
            sort YEAR PID
        tempfile fatherdata
            save `fatherdata'

    * Information on children of mothers (RB230) ****************

          use `data', clear

          * Education status
            gen EDUC = 0
              replace EDUC = 1 if ACTIVITY==1 & AGE<35
              label var EDUC "in education an younger than 35"

          * Keep data for those persons whose mother is in the survey
            keep EDUC RB230 HHID YEAR
            drop if RB230==.  // drop if mother is not living in the same household
        rename RB230 PID // mothers ID becomes ID - for merging

      * Identify siblings (children of a certain person)
        egen SIBLING = group(YEAR HHID PID)  // group all children of a person who
live in the HH
        sort SIBLING

      * Number of children in education
            by SIBLING: egen EDUCHILD=total(EDUC==1)
              label var EDUCHILD "number of children in education"

      * Number of children
            bysort YEAR HHID SIBLING: egen NCHILD=count(PID)
              label var NCHILD "number of children"

          * Select "representative"
```

```
        egen SIBLINGTAG = tag(SIBLING)
        keep if SIBLINGTAG==1

          * Save child dataset
            sort YEAR PID
        tempfile motherdata
            save `motherdata'

    * Merge datasets ****************************

      use `data', clear

          sort YEAR PID
          merge YEAR PID using `fatherdata', update

          drop _merge

          sort YEAR PID
          merge YEAR PID using `motherdata', update

          drop _merge

    * Distribute family benefits *****************
      // Family benefits are distributed to parents with children in education
          // inverse to their labour income; otherwise to all adults in equal shares

          * Share of primary income within couples
              bysort YEAR HHID COUPLEID: egen inc_couple = sum(PYG) if COUPLEID!=.
              gen incshare_couple = PYG/inc_couple
                  replace incshare_couple = -incshare_couple if incshare_couple<0
          // Negative income is treated as if it were positive because it also
indicates labour market activity
                  // Family benefits should be assigned to the person who carries
out most of the childcare

          * Number of children in education is the maximum within couples, assuming
that couples care together for children
            * even if one of the partners is not father/mother
              bysort YEAR HHID COUPLEID: egen NEDUCHILD = max(EDUCHILD) if
COUPLEID!=.
              replace NEDUCHILD = EDUCHILD if COUPLEID==.

          * Children are distributed within couples according to inverse of income
share
            // the indicator serves as weight to distribute family benefits
                  replace NEDUCHILD = NEDUCHILD*(1-incshare_couple) if
incshare_couple!=.

          * Generation of weights summing up to 1 within the household
              bysort YEAR HHID: egen NEDUCHILDHH = sum(NEDUCHILD)
```

```
                  replace NEDUCHILD = NEDUCHILD/NEDUCHILDHH

          * If children cannot be identified the family transfers are assigned to
all adults
              drop NEDUCHILDHH
              bysort YEAR HHID: egen NEDUCHILDHH = sum(NEDUCHILD)
                  replace NEDUCHILD = 1/NADULT if ADULT==1 & NEDUCHILDHH==0

          gen HY050Gp = HY050G*NEDUCHILD
            replace HY050Gp = 0 if HY050Gp==.
                label var HY050Gp "Individualized family benefits, gross"


          gen HY050Np = HY050N*NEDUCHILD
          replace HY050Np = 0 if HY050Np==.
          // gen HY050Np = 0 // alternative assignment to all households
          //replace HY050Np = HY050N/NADULT if ADULT==1
          label var HY050Np "Individualized family benefits, net"

          * Control if values add up
            bysort YEAR HHID: egen cHY050Gp = sum(HY050Gp)
            bysort YEAR HHID: egen cHY050Np = sum(HY050Np)
            browse YEAR HHID AGE SEX HY050N HY050Gp cHY050Np cHY050Gp HY050Np

                sum HY050N cHY050N


  **************************************************
  * Individualize imputed rent
  **************************************************

    * Imputed rent assigned to the couple responsible for accomodation
      replace ACCOMODATION=1 if ACCOMODATION_P==1
          bysort YEAR HHID: egen NACCOMOD = total(ACCOMODATION==1)

    * Imputed rent is calculated as imputed rent less interest payments on mortgage
          gen HYRGp = (HY030G - HY100G)/NACCOMOD
            label var HYRGp "Individualized imputed rent"

          gen HYRNp = (HY030N - HY100N)/NACCOMOD
          // gen HYRNp = (HY030N - HY100N)/NADULT if ADULT==1 // alternative
assignment to all adults
            label var HYRNp "Individualized imputed rent"

    * We do not allow negative imputed rents
    // There are even persons not owning the home but paying interest on mortgage
          replace HYRGp = 0 if HYRGp<0 | ACCOMODATION==0
          replace HYRNp = 0 if HYRNp<0 | ACCOMODATION==0

    * If net values not available, use gross
```

```
          bysort YEAR: egen RNSUM = total(HYRNp)
            replace HYRNp = HYRGp if RNSUM==0
                drop RNSUM

    * Control if it adds up to total
         bysort YEAR HHID: egen cHYRNp = sum(HYRNp)


  **************************************************
  * Income of persons below 16
  **************************************************

    * Number of children in older child ages (most income of children is generated
by 15 year olds)
      bysort YEAR HHID: egen NCHILD16 = total(AGE==16)
      bysort YEAR HHID: egen NCHILD15 = total(AGE==15)
      bysort YEAR HHID: egen NCHILD14 = total(AGE<15 & AGE>6)

    gen HYCHGp = 0
    gen HYCHNp = 0

    * If possible income is assignes to 15 year old (they are most likely to receive
income)
      replace HYCHGp = HY110G/NCHILD15 if NCHILD15>=1 & AGE==15
      replace HYCHNp = HY110N/NCHILD15 if NCHILD15>=1 & AGE==15

    * If there is no 15 year old, also younger children above age 6 are considered
to receive income
      replace HYCHGp = HY110G/NCHILD14 if NCHILD15==0 & NCHILD14>=1 & AGE<15 & AGE>6
      replace HYCHNp = HY110N/NCHILD14 if NCHILD15==0 & NCHILD14>=1 & AGE<15 & AGE>6

    * It is possible that 16 year old receive child-income if no younger children
are in the HH
      replace HYCHGp = HY110G/NCHILD16 if NCHILD15==0 & NCHILD14==0 & NCHILD16>=1  &
AGE==16
      replace HYCHNp = HY110N/NCHILD16 if NCHILD15==0 & NCHILD14==0 & NCHILD16>=1  &
AGE==16

    * If no children older than 6, we assume that the value for child income is a
mistake ad set it to zero
      replace HYCHGp = 0 if NCHILD15==0 & NCHILD14==0 & NCHILD16==0
      replace HYCHNp = 0 if NCHILD15==0 & NCHILD14==0 & NCHILD16==0

    * Control if it adds up to total
      bysort YEAR HHID: egen cHYCHGp = sum(HYCHGp)
          bysort YEAR HHID: egen cHYCHNp = sum(HYCHNp)


  **************************************************
  * Household income components assigned to adults
```

```
*     p indicates personalzed components of household income
**************************************************

  * Asset income
    gen HYAGp = 0
      replace HYAGp = (HY040G + HY090G - HY120G)/NADULT if ADULT==1
    // Income from rent of property, from investments in incorporated businesses
less taxes on wealth

    gen HYANp = 0
      replace HYANp = (HY040N + HY090N - HY120N)/NADULT if ADULT==1


  * Public transfers at household level

    gen HYTGGp = 0
      replace HYTGGp = (HY060G + HY070G)/NADULT if ADULT==1
      // social exclusion, housing
          replace HYTGGp = HYTGGp + HY050Gp

    gen HYTGNp = 0
      replace HYTGNp = (HY060N + HY070N)/NADULT if ADULT==1
      replace HYTGNp = HYTGNp + HY050Np

  * Private transfers at household level (received - paid)

    gen HYTFGp = 0
      replace HYTFGp = (HY080G - HY130G)/NADULT if ADULT==1
      // social, exclusion, housing, inter-household transfers

    gen HYTFNp = 0
      replace HYTFNp = (HY080N - HY130N)/NADULT if ADULT==1

  * Total household level transfers

        gen HYTGp = HYTFGp + HYTGGp
        gen HYTNp = HYTFNp + HYTGNp


**************************************************
* Total personalized income
**************************************************

* Labour income
  gen YLG =  PY010G + PY021G + PY050G + HYCHGp
  gen YLN =  PY010N + PY021N + PY050N + HYCHNp
  replace YLG =  PY010G + PY020G + PY050G + HYCHGp if YEAR<2006
  replace YLN =  PY010N + PY020N + PY050N + HYCHNp if YEAR<2006
  gen YEMPLN =  PY010N + PY021N + HYCHNp
  gen YEMPLG =  PY010G + PY021G + HYCHGp
```

```
  replace YEMPLN =  PY010N + PY020N + HYCHNp
  replace YEMPLG =  PY010G + PY020G + HYCHGp
    // PY010G, PY020G: labour income, cash, in-kind
    // PY050: income form self-employment

* Asset income
  gen YAG = HYAGp
  gen YAN = HYANp
  replace YAG = HYAGp + PY080G if YEAR>=2011
  replace YAN = HYANp + PY080N if YEAR>=2011
    // PY080: income from private pension plans

* Imputed rent
  gen YRG = HYRGp
  gen YRN = HYRNp

* Total primary income (labour + asset income, no transfers)
  gen YPG = YLG + YAG // + YRG
  gen YPN = YLN + YAN // + YRN

      // HYR is defined as the positive values of HY030 - HY100
  // HYCH is HY110 if there is a child in the household
      // HYAG is HY040G + HY090G - HY120G

* Comments:
  // In Austria PY021 (company car) is always 0

* Public transfers
  gen YTGG = PY090G + PY100G + PY110G + PY120G + PY130G + PY140G + HYTGGp
  gen YTGN = PY090N + PY100N + PY110N + PY120N + PY130N + PY140N + HYTGNp

  gen YTGGOLD = PY100G + PY110G + PY120G + PY130G
  gen YTGNOLD = PY100N + PY110N + PY120N + PY130N

  gen YTGGOTH = PY090G + PY140G + HYTGGp
  gen YTGNOTH = PY090N + PY140N + HYTGNp

* Private transfers
  gen YTFG = HYTFGp
  gen YTFN = HYTFNp

* Total transfers
  gen YTG = YTGG + YTFG
  gen YTN = YTGN + YTFN
    // PY090 Unemployment benefits
        // PY100 Old age benefits
        // PY110 Survivor benefits
        // PY120 Sickness benefits
        // PY130 Disability benefits
        // PY140 Education related allowances
```

```
          // YTGN is HY060N + HY070N, YTFN is HY080N - HY130N
          //  = social exclusion n.e.c., housing, inter-household received,
inter-household paid

  * Total income (primary income + transfers)
    gen YG = YPG + YTG // + YRG
    gen YN = YPN + YTN // + YRN

        gen YN1 = YN + YRN
          label var YN1 "Net income incl. imputed rent"
    gen YG1 = YG + YRG
          label var YG1 "Gross income incl. imputed rent"

  * Compare with household income
    bysort YEAR HHID: egen YGh = sum(YG)
    bysort YEAR HHID: egen YNh = sum(YN)

  * Imputed rent at household level
    bysort YEAR HHID: egen YRGh = sum(YRG)
    bysort YEAR HHID: egen YRNh = sum(YRN)

  * Taxes
    capture gen TAX = HY140N
      replace TAX = 0 if CNT2=="SE" | CNT2=="PL" | CNT2=="BG" | CNT=="CZ" |
CNT2=="IE" | CNT2=="LV" | CNT2=="RO" | CNT2=="SI"
      replace TAX = 0 if CNT2=="ES" & YEAR<=2006
      replace TAX = 0 if CNT2=="PL" & YEAR==2004

    replace YGh = YGh - HY140G // + HY110G
    replace YNh = YNh - HY140N - HY145N // + HY110N
        gen dYh = YGh - YNh
    // HY140 tax on income and social contributions

        gen YGh_diff = HY020 - YGh



  ****************************************************
  * Aggregate indicators based on individual income
  ****************************************************

  * Median individual income

        gen YN1MED = .
          label var YN1MED "Median, individual income"
                sum YEAR
                forvalues y = `r(min)'/`r(max)' {
                  sum YN1 [aw=WEIGHT] if YEAR==`y' & AGE>=20, det
              replace YN1MED=`r(p50)' if YEAR==`y'
                }
```

```
        gen YG1MED = .
          label var YG1MED "Median, individual income"
                sum YEAR
                forvalues y = `r(min)'/`r(max)' {
                  sum YG1 [aw=WEIGHT] if YEAR==`y' & AGE>=20, det
              replace YG1MED=`r(p50)' if YEAR==`y'
                }

        gen YNMED = .
          label var YNMED "Median, individual income"
                sum YEAR
                forvalues y = `r(min)'/`r(max)' {
                  sum YN [aw=WEIGHT] if YEAR==`y' & AGE>=20, det
              replace YNMED=`r(p50)' if YEAR==`y'
                }

        gen YGMED = .
          label var YGMED "Median, individual income"
                sum YEAR
                forvalues y = `r(min)'/`r(max)' {
                  sum YG [aw=WEIGHT] if YEAR==`y' & AGE>=20, det
              replace YGMED=`r(p50)' if YEAR==`y'
                }

  * Share of Persons above median income

    gen YN1MEDPLUS = 0
          replace YN1MEDPLUS = 1 if YN1>=YN1MED

    gen YG1MEDPLUS = 0
          replace YG1MEDPLUS = 1 if YG1>=YG1MED


  ***************
  * Real income
  ***************

    sort CNT2 YEAR
        merge CNT2 YEAR using hicp
        drop if _merge!=3

        gen HICP17t = HICP if YEAR==2017
        bysort CNT2: egen HICP17 = mean(HICP17t)
        replace HICP = HICP/HICP17*100

    local incomes "YN1 YN YNMED YN1MED YG1 YG YGMED YG1MED YPN YTN YTGN YTFN YTGG
YLN YEMPLN YAN YRN YPG YTG YLG YAG YRG"
    foreach var of local incomes {
      gen R`var' = `var'/HICP*100
```

```
    }

  save silc_allyears_`x', replace

}

/*
*************************************************
* 0. Aggregate income (for control with ESA data)
*************************************************

foreach x in $country {
    use data/silc`x'0_3, clear

    local indic "RYN1 RYRN RYTGN RYTFN RYLN RYAN RYEMPLN"
    keep YEAR CNT2 `indic' WEIGHT
        gen ONE = 1
    collapse (sum) `indic' ONE [pw=WEIGHT], by(CNT2 YEAR)
        keep if YEAR==2008 | YEAR==2017
        tempfile aggr`x'
        save `aggr`x''
}

use `aggrAT'
gen temp=1
foreach x in $country {
  append using `aggr`x''
}
drop if temp==1
drop temp
sort YEAR CNT2
browse YEAR CNT2 RYN1 RYTGN RYEMPLN
*/



*********************************************************************************************
************************************************
* 2. Descriptive statistics: age-specific means
*********************************************************************************************
************************************************


  ******************************************************************
  * Calculate mean and confidence intervals for the mean
  ******************************************************************

  local indic "RYN1" // indicator
  local lstage "AGEGR20" // life stage variable (AGE or LIFESTAGE)
```

```
  local stat "mean" // statistic: mean, proportion, ratio, total

  foreach x in $country {

  *******************
  * Total (no age)
  *******************

  use silc_allyears_`x', clear
  replace PSU = HHID
  drop if AGEGR20==0

    keep YEAR CNT2 `indic' PSU WEIGHT STRATA

    gen SUBPOP = YEAR
    svyset PSU [pw=WEIGHT], strata(STRATA)

    tempfile silc
    save `silc', replace

    sum YEAR
    local min = `r(min)'
    local max = `r(max)'

    forvalues i = `r(min)'/`r(max)' {
      use `silc', clear
      svy, subpop(if SUBPOP==`i'): `stat'  `indic'
      // matrix list r(table) // list content of matrix
      xsvmat, from(r(table)') rownames(rname) names(col) norestore // convert matrix
to output data

      gen year =`i'
      tempfile t`i'
      save `t`i'', replace
    }

    use `t`min'', clear
    local min = `min'+1
    forvalues i = `min'/`max' {
      append using `t`i''
    }

        gen SEX = 0
        gen `lstage' = 99 // total adult population

    tempfile `x't
        save ``x't'


  *******************
```

```
* Total by age (m+f)
*******************

  use silc_allyears_`x', clear
  drop if AGEGR20==0
  replace PSU = HHID

  keep YEAR CNT2 `indic' `lstage' PSU WEIGHT STRATA

  gen SUBPOP = YEAR
  svyset PSU [pw=WEIGHT], strata(STRATA)

  tempfile silc
  save `silc', replace

  sum YEAR
  local min = `r(min)'
  local max = `r(max)'

  forvalues i = `r(min)'/`r(max)' {
    use `silc', clear
    svy, subpop(if SUBPOP==`i'): `stat'  `indic' , over(`lstage')
    matrix list r(table)
    xsvmat, from(r(table)') rownames(rname) names(col) norestore

    gen year =`i'
    tempfile t`i'
    save `t`i'', replace
  }

  use `t`min'', clear
  local min = `min'+1
  forvalues i = `min'/`max' {
    append using `t`i''
  }

      gen SEX = 0

      tempfile `x'
      save ``x''

*******************
* Men by age
*******************

  use silc_allyears_`x', clear
      keep if SEX==1
  drop if AGEGR20==0
  replace PSU = HHID
```

```
  keep YEAR CNT2 `indic' `lstage' PSU WEIGHT STRATA

  gen SUBPOP = YEAR
  svyset PSU [pw=WEIGHT], strata(STRATA)

  tempfile silc
  save `silc', replace

  sum YEAR
  local min = `r(min)'
  local max = `r(max)'

  forvalues i = `r(min)'/`r(max)' {
    use `silc', clear
    svy, subpop(if SUBPOP==`i'): `stat'  `indic' , over(`lstage')
    matrix list r(table)
    xsvmat, from(r(table)') rownames(rname) names(col) norestore

    gen year =`i'
    tempfile t`i'
    save `t`i'', replace
  }

  use `t`min'', clear
  local min = `min'+1
  forvalues i = `min'/`max' {
    append using `t`i''
  }

      gen SEX = 1

      tempfile `x'm
      save ``x'm'

*******************
* Women by age
*******************

  use silc_allyears_`x', clear
      keep if SEX==2
  drop if AGEGR20==0
  replace PSU = HHID

  keep YEAR CNT2 `indic' `lstage' PSU WEIGHT STRATA

  gen SUBPOP = YEAR
  svyset PSU [pw=WEIGHT], strata(STRATA)

  tempfile silc
  save `silc', replace
```

```
    sum YEAR
    local min = `r(min)'
    local max = `r(max)'

    forvalues i = `r(min)'/`r(max)' {
      use `silc', clear
      svy, subpop(if SUBPOP==`i'): `stat'  `indic' , over(`lstage')
      matrix list r(table)
      xsvmat, from(r(table)') rownames(rname) names(col) norestore

      gen year =`i'
      tempfile t`i'
      save `t`i'', replace
    }

    use `t`min'', clear
    local min = `min'+1
    forvalues i = `min'/`max' {
      append using `t`i''
    }
        gen SEX = 2

        * Merge sex-specific data

        append using ``x't'
        append using ``x''
        append using ``x'm'

    rename b VAL
    rename se SE
    rename ll CI95L
    rename ul CI95U
    rename year YEAR
    gen CNT2 = "`x'"
    gen STAT = "`stat'"
    gen INDIC = "`indic'"

          replace AGEGR20 = 2 if rname=="c.`indic'@2.`lstage'"
          replace AGEGR20 = 4 if rname=="c.`indic'@4.`lstage'"
          replace AGEGR20 = 6 if rname=="c.`indic'@6.`lstage'"
    label val AGEGR20 AGEGR20

        keep YEAR CNT2 `lstage' SEX INDIC STAT VAL SE CI95L CI95U
        sort CNT2 YEAR `lstage' SEX

        tempfile `x'
        save ``x''

}
```

```
********************************
* Merge country means
********************************

  use `AT', clear
  gen temp = 1
  foreach x in $country {
    append using ``x''
  }
  drop if temp==1
  drop temp

  save age3_`indic'_mean, replace

  /*
  * Data for paper
    gen SEX = 0
    order SEX CNT2 YEAR AGEGR20 VAL SE CI95L CI95U INDIC        STAT
    sort SEX YEAR AGEGR20 CNT2
    keep if YEAR==2008 | YEAR==2017
  */



*********************************************************************************
**********************************************
* 3. Descriptive statistics: Percentiles of age-specific means
*********************************************************************************
**********************************************

  local indic "RYN1" // indicator
  local lstage "AGEGR20"

  foreach x in $country {

        use silc_allyears_`x', clear

    keep YEAR `lstage' WEIGHT STRATA PSU `indic' AGE SEX
    svyset PSU [pw=WEIGHT], strata(STRATA) singleunit(scaled)
        keep if AGEGR20>0
        tempfile silc
    save `silc'

  *****************
  * Total, no age
  *****************

  use `silc', clear
```

```
  sum YEAR
  local yearmin = `r(min)'
  local yearmax = `r(max)'

  forvalues y = `yearmin'/`yearmax' {
          display `y'
      use `silc', clear
      keep YEAR WEIGHT STRATA PSU `indic'
          keep if YEAR==`y'
      epctile `indic', percentiles(25 50 75) svy
      // matrix list r(table)
      xsvmat, from(r(table)') rownames(rname) names(col) norestore

      gen YEAR =`y'
      gen `lstage' = 99

      tempfile t`y'
      save `t`y'', replace
    }

  use `t`yearmin'', clear
  gen DROP = 1
  forvalues y = `yearmin'/`yearmax' {
     append using `t`y''
    }

  drop if DROP==1
     gen SEX = 0

  tempfile `x't
     save ``x't'


******************
* Total (m+f)
******************

  use `silc', clear
  sum YEAR
  local yearmin = `r(min)'
  local yearmax = `r(max)'
  sum `lstage'
  local submin = `r(min)'
  local submax = `r(max)'



  forvalues y = `yearmin'/`yearmax' {
    forvalues s = `submin'(2)`submax' {
           display("Hello")
```

```
              display(`s')
              display(`y')
              count
              display("Here we are")
    use `silc', clear
            keep if YEAR==`y' & `lstage'==`s'
                count
                epctile `indic', percentiles(25 50 75) svy
        // matrix list r(table)
        xsvmat, from(r(table)') rownames(rname) names(col) norestore

        gen YEAR =`y'
        gen `lstage' =`s'

        tempfile t`y'`s'
        save `t`y'`s'', replace
    }
  }



  use `t`yearmin'`submin'', clear
  gen DROP = 1
  forvalues y = `yearmin'/`yearmax' {
    forvalues s = `submin'(2)`submax' {
      append using `t`y'`s''
    }
  }
  drop if DROP==1
      gen SEX = 0

  tempfile `x'
      save ``x''

******************
* Men
******************

  use `silc', clear
      keep if SEX==1
  sum YEAR
  local yearmin = `r(min)'
  local yearmax = `r(max)'
  sum `lstage'
  local submin = `r(min)'
  local submax = `r(max)'

  forvalues y = `yearmin'/`yearmax' {
    forvalues s = `submin'(2)`submax' {
        use `silc', clear
```

```
            keep if YEAR==`y' & `lstage'==`s'
        epctile `indic', percentiles(25 50 75) svy
        // matrix list r(table)
        xsvmat, from(r(table)') rownames(rname) names(col) norestore

        gen YEAR =`y'
        gen `lstage' =`s'

        tempfile t`y'`s'
        save `t`y'`s'', replace
    }
  }

  use `t`yearmin'`submin'', clear
  gen DROP = 1
  forvalues y = `yearmin'/`yearmax' {
    forvalues s = `submin'(2)`submax' {
      append using `t`y'`s''
    }
  }

  drop if DROP==1
      gen SEX = 1

  tempfile `x'm
      save ``x'm'


******************
* Women
******************

  use `silc', clear
      keep if SEX==2
  sum YEAR
  local yearmin = `r(min)'
  local yearmax = `r(max)'
  sum `lstage'
  local submin = `r(min)'
  local submax = `r(max)'

  forvalues y = `yearmin'/`yearmax' {
    forvalues s = `submin'(2)`submax' {
        use `silc', clear
            keep if YEAR==`y' & `lstage'==`s'
        epctile `indic', percentiles(25 50 75) svy
        // matrix list r(table)
        xsvmat, from(r(table)') rownames(rname) names(col) norestore

        gen YEAR =`y'
```

```
          gen `lstage' =`s'

          tempfile t`y'`s'
          save `t`y'`s'', replace
        }
      }

      use `t`yearmin'`submin'', clear
      gen DROP = 1
      forvalues y = `yearmin'/`yearmax' {
        forvalues s = `submin'(2)`submax' {
          append using `t`y'`s''
        }
      }
      drop if DROP==1
          gen SEX = 2

      * Merge sex-specific data

          append using ``x't'
          append using  ``x''
          append using  ``x'm'

          * Name variables

      rename b VAL
      rename rname STAT
      rename se SE
      rename ll CI95L
      rename ul CI95U
      gen CNT2 = "`x'"
      gen INDIC = "`indic'"

          keep CNT2 YEAR `lstage' SEX STAT INDIC VAL SE CI95L CI95U

          tempfile `x'
          save ``x''

  }

    ************************************
    * Merge countries
    ************************************

    use `AT', clear
    gen temp = 1
    foreach x in $country {
      append using ``x''
    }
    drop if temp==1
```

```
  drop temp

  save age3_`indic'_perc, replace


************************************************************************************
**************************************
* 4. Means and percentiles: Copy and paste to Excel file
************************************************************************************
**************************************

local indic RYN1
  use age3_`indic'_perc, clear
        append using age3_`indic'_mean

  save age3_`indic', replace

  *  Data for paper: Copy and paste to Tables_age_specific_income_trends

  * Means and percentiles by age and sex
    use age3_RYN1, replace
    order SEX CNT2 YEAR AGEGR20 VAL CI95L CI95U INDIC STAT
    sort YEAR AGEGR20 CNT2
    browse if (YEAR==2008 | YEAR==2017) & SEX==1 & STAT=="mean"  // For Table 3:
Tab3_mean_inc_gender
    browse if (YEAR==2008 | YEAR==2017) & SEX==2 & STAT=="mean"  // For Table 3:
Tab3_mean_inc_gender
    browse if (YEAR==2008 | YEAR==2017) & SEX==0 & STAT=="mean"  // For Table 2:
Tab2_mean_inc_gender
    browse if (YEAR==2008 | YEAR==2017) & SEX==0 & STAT=="p25"   // For Table 6
    browse if (YEAR==2008 | YEAR==2017) & SEX==0 & STAT=="p50"   // For Table 5
    browse if (YEAR==2008 | YEAR==2017) & SEX==0 & STAT=="p75"   // For Table 7



************************************************************************************
*****************************************************
* 4. Decompositiopn of income changes
************************************************************************************
*****************************************************

local indic "RYN1 RYRN RYTGN RYTFN RYLN RYAN EMPL RYOTH"
local lstage "AGEGR20"

local country AT

foreach x in $country {

  use silc_allyears_`x', clear
  keep if AGEGR20==2 | AGEGR20==4 | AGEGR20==6
```

```
 * Employment status
       egen PARTT = rowtotal(PL074 PL076)  // Number of months working parttime
       egen FULLT = rowtotal(PL073 PL075)
       egen RETIR = rowtotal(PL085 PL086) // retirement, disability
   *browse YEAR HHID SEX AGE ACTIVITY YLN PARTT FULLT RETIR

       gen EMPL = 0
       tempvar EMPLMONTHS
       gen `EMPLMONTHS' = (PARTT + FULLT)/12
       replace EMPL = `EMPLMONTHS'

       * If labour is above threshold and infor an employment missing
         browse YEAR AGE HHID YLN PARTT FULLT EMPL
         replace EMPL = 1 if YLN>YNMED & EMPL==0 & YEAR>=2008
         replace EMPL = 0.5 if YLN>YNMED/3 & EMPL==0 & YEAR>=2008
         replace EMPL = 0 if YLN<500 & YEAR>=2008 & EMPL>0.1

 * Income category other
   gen RYOTH = RYTFN + RYAN + RYRN

 * Population
   gen POP = 1

*******************
* Total (not lifestage-specific)
*******************

 preserve
 collapse (mean) `indic' (sum) POP [pw=WEIGHT], by(CNT2 YEAR)
 gen STAT = "MEAN"
 gen SEX = 0
 gen `lstage' = 99
 tempfile ttotal
 save `ttotal'
 restore

 preserve
 collapse (mean) `indic' (sum) POP [pw=WEIGHT], by(CNT2 YEAR SEX)
 gen STAT = "MEAN"
 gen `lstage' = 99
 tempfile stotal
 save `stotal'
 restore

 preserve
 collapse (mean) `indic' (sum) POP [pw=WEIGHT], by(CNT2 YEAR `lstage')
 gen STAT = "MEAN"
 gen SEX = 0
 tempfile total
```

```
  save `total'
  restore

  preserve
  keep if SEX==1
  collapse (mean) `indic' (sum) POP [pw=WEIGHT], by(CNT2 YEAR SEX `lstage')
  gen STAT = "MEAN"
  tempfile men
  save `men'
  restore

  keep if SEX==2
  collapse (mean) `indic' (sum) POP [pw=WEIGHT], by(CNT2 YEAR SEX `lstage')
  gen STAT = "MEAN"

  append using `men'
  append using `total'
  append using `stotal'
  append using `ttotal'

  save mean_`x', replace
}

  * Combine data for all countries

    use mean_AT, clear
    gen TEMP = 1
    foreach x in $country {
      append using mean_`x'
    }
    drop if TEMP==1
    drop TEMP

  save decomp_SILC, replace


  ************************************************
  * Calculate difference 2008 - 2017
  ************************************************

  use decomp_SILC, clear

    local variables "RYN1 RYRN RYTGN RYTFN RYLN RYAN RYOTH EMPL" // EMPLy
WORKHOURSempl WAGEHOUR"
    local lstage "AGEGR20"

        tempfile data
        sort CNT2 SEX `lstage'
        save `data'
```

```
        keep if YEAR==2008
        foreach var of local variables {
          rename `var' `var'08
        }
        sort CNT2 SEX `lstage'
        tempfile data08
        save `data08'

        use `data'
        sort CNT2 SEX `lstage'
        merge CNT2 SEX `lstage' using `data08'
        drop _merge

  * Changes relative to 2008 income
    local variables "RYN1 RYRN RYTGN RYTFN RYLN RYAN RYOTH" // EMPLy WORKHOURSempl
WAGEHOUR"
    foreach var of local variables {
          gen D`var' = (`var' - `var'08)/RYN108
        }

  save decomp_SILC_diff, replace


  *****************************************************
  * Decompose changes in income men and women
  *****************************************************

  use decomp_SILC_diff, clear

  keep if YEAR==2008 | YEAR==2017
  replace DRYN1 = . if YEAR==2008
  gen POP08t = POP if SEX==0 & YEAR==2008
  gen POP08f = POP if SEX==2 & YEAR==2008
  gen POP08m = POP if SEX==1 & YEAR==2008

  gen POP17t = POP if SEX==0 & YEAR==2017
  gen POP17f = POP if SEX==2 & YEAR==2017
  gen POP17m = POP if SEX==1 & YEAR==2017

  gen D = DRYN1 if SEX==0
  gen Dm = DRYN1 if SEX==1
  gen Df = DRYN1 if SEX==2

  collapse POP08t POP08m POP08f POP17t POP17m POP17f D Dm Df, by(CNT2 AGEGR20)
  gen DIFF = Dm*(POP17m+POP08m)/(POP17t+POP08t) + Df*(POP17f+POP08f)/(POP17t+POP08t)


***********************************************************
* Decompose changes in income by employment status
***********************************************************
```

```
  use decomp_SILC_diff, clear

  * Decomposition labour income
    gen DRYLNp = log(RYLN) - log(RYLN08)
    gen DEMPLp = log(EMPL) - log(EMPL08)
        gen DWAGEp = DRYLNp - DEMPLp

        gen DEMPL = DRYLN*DEMPLp/DRYLNp
        gen DWAGE = DRYLN*DWAGEp/DRYLNp

  * Decomposition transfer income
    gen DRYTGNp = log(RYTGN) - log(RYTGN08)
    gen DNEMPLp = log(1-EMPL) - log(1-EMPL08)
        gen DRECp = DRYTGNp - DNEMPLp

        gen DNEMPL = DRYTGN*DNEMPLp/DRYTGNp
        gen DREC = DRYTGN*DRECp/DRYTGNp


  *********************************************************
  * Table for paper; Table 4
  *********************************************************

  sort CNT2 YEAR SEX AGEGR20
  order CNT2 YEAR SEX AGEGR20 RYN1 RYLN RYTGN RYOTH DRYN1 DRYLN DEMPL DWAGE DRYTGN
DNEMPL DREC DRYOTH RYN108

  browse if YEAR==2017 & SEX==0


**********************************************************************************
*****************************************
* 6. Graphs of age-specific income changes, Fig. 1
**********************************************************************************
*****************************************

    local variables "DRYLN DEMPL DWAGE DRYTGN DNEMPL DREC DRYOTH"
        browse YEAR AGEGR20 `variables'
    foreach var of local variables {
          gen `var'neg = 0
          replace `var'neg = `var'*100 if `var'<0
          gen `var'pos = 0
          replace `var'pos = `var'*100 if `var'>=0
        }
        replace DRYN1 = DRYN1*100

        gen DRYN0pos = DEMPLpos + DWAGEpos + DNEMPLpos + DRECpos + DRYOTHpos
        gen DRYN1pos = DWAGEpos + DNEMPLpos + DRECpos + DRYOTHpos
        gen DRYN2pos = DNEMPLpos + DRECpos + DRYOTHpos
```

```
        gen DRYN3pos = DRECpos + DRYOTHpos
        gen DRYN4pos = DRYOTHpos

        gen DRYN0neg = DEMPLneg + DWAGEneg + DNEMPLneg + DRECneg + DRYOTHneg
        gen DRYN1neg = DWAGEneg + DNEMPLneg + DRECneg + DRYOTHneg
        gen DRYN2neg = DNEMPLneg + DRECneg + DRYOTHneg
        gen DRYN3neg = DRECneg + DRYOTHneg
        gen DRYN4neg = DRYOTHneg

  label drop AGEGR20
  gen AGEVAR = 1 if AGEGR20==2
  replace AGEVAR = 2 if AGEGR20==4
  replace AGEVAR = 3 if AGEGR20==6
  replace AGEVAR = 4.6 if AGEGR20==99

  keep if YEAR==2017


*******************************
* Austria
*******************************

  local cnt "AT"
  local sex = 0
  local bw = 0.6
  local yscl = -6
  local yscu = 20
  local ylabl = -5
  local ylablu = 20
  local xs = 8
  local ys = 6
  local lc = "gs2"

  graph twoway bar DRYN0pos AGEVAR if SEX==`sex' & CNT2=="`cnt'", barw(`bw') col("0
29 74") lcol(`lc')  ///
      || bar DRYN1pos AGEVAR if SEX==`sex' & CNT2=="`cnt'", barw(`bw') col("48 122
133") lcol(`lc') ///
          || bar DRYN2pos AGEVAR if SEX==`sex' & CNT2=="`cnt'", barw(`bw') col("255
180 0") lcol(`lc') ///
          || bar DRYN3pos AGEVAR if SEX==`sex' & CNT2=="`cnt'", barw(`bw')
col("orange") lcol(`lc') ///
          || bar DRYN4pos AGEVAR if SEX==`sex' & CNT2=="`cnt'", barw(`bw') col("190
190 190") lcol(`lc') ///
      || bar DRYN0neg AGEVAR if SEX==`sex' & CNT2=="`cnt'", barw(`bw') col("0 29
74") lcol(`lc') ///
      || bar DRYN1neg AGEVAR if SEX==`sex' & CNT2=="`cnt'", barw(`bw') col("48 122
133") lcol(`lc') ///
          || bar DRYN2neg AGEVAR if SEX==`sex' & CNT2=="`cnt'", barw(`bw') col("255
180 0") lcol(`lc') ///
          || bar DRYN3neg AGEVAR if SEX==`sex' & CNT2=="`cnt'", barw(`bw')
```

```
col("orange") lcol(`lc') ///
          || bar DRYN4neg AGEVAR if SEX==`sex' & CNT2=="`cnt'", barw(`bw') col("190
190 190") lcol(`lc') ///
          || scatter DRYN1 AGEVAR if SEX==`sex' & CNT2=="`cnt'", col(black)  ///
          yline(0, lcol(gs5)) ///
          graphregion(margin(l-3 r-2 t+0 b-3) color(white)) ///
          plotregion(margin(tiny)) ///
      title("`cnt', men and women", size(med) margin(tiny)) ///
     ytitle("Income change 2008 - 2017 in %", size(medsmall)) ///
          ysc(r(`yscl' `yscu')) xsc(r(0.5 3.5)) ///
          ylabel(`ylabl'(5)`ylablu') ///
          xlabel(1 "20-39" 2 "40-59" 3 "60+" 4.6 "Total", labsize(medsmall)) ///

          xtitle("", margin(0 0 0 2)) ///
      legend(cols(2) label(1 "Employment rates (l)") label(2 "Income per employed
(yl)") label(3 "Share of beneficiaries (n)") ///
          label(4 "Benefits per beneficiary (yb)") label(5 "Other income (YO)")
label(11 "Total change") order(1 2 3 4 5 11) rowgap(*0.8) ///
          bmargin(small) size(small) ring(1) position(11) symx(*0.5))
      graph display, xsize(`xs') ysize(`ys')
          graph save $graphslocation/dec_`cnt'_`sex', replace
          graph export $graphslocation/dec_`cnt'_`sex'.tif, replace
          graph export $graphslocation/dec_`cnt'_`sex'.png, replace


  gen D0pos = DRYLNpos + DRYTGNpos + DRYOTHpos
  gen D1pos = DRYTGNpos + DRYOTHpos
  gen D2pos = DRYOTHpos

  gen D0neg = DRYLNneg + DRYTGNneg + DRYOTHneg
  gen D1neg = DRYTGNneg + DRYOTHneg
  gen D2neg = DRYOTHneg

  local cnt "AT"
  local sex = 0
  local bw = 0.6
  local yscl = -6
  local yscu = 12
  local ylabl = -5
  local ylablu = 12
  local xs = 8
  local ys = 5

  graph twoway bar D0pos AGEVAR if SEX==`sex' & CNT2=="`cnt'", barw(`bw') col("50 80
100")   ///
      || bar D1pos AGEVAR if SEX==`sex' & CNT2=="`cnt'", barw(`bw') col("230 120 0")
///
          || bar D2pos AGEVAR if SEX==`sex' & CNT2=="`cnt'", barw(`bw') col("230 210
80") ///
      || bar D0neg AGEVAR if SEX==`sex' & CNT2=="`cnt'", barw(`bw') col("50 80 100")
```

```
///
      || bar D1neg AGEVAR if SEX==`sex' & CNT2=="`cnt'", barw(`bw') col("230 120 0")
///
          || bar D2neg AGEVAR if SEX==`sex' & CNT2=="`cnt'", barw(`bw') col("230 210
80") ///
          || scatter DRYN1 AGEVAR if SEX==`sex' & CNT2=="`cnt'", col(black)  ///
          yline(0, lcol(gs5)) ///
          graphregion(margin(l-3 r-2 t+0 b-3) color(white)) ///
          plotregion(margin(tiny)) ///
     ytitle("Einkommensänderung 2008 - 2017 in %", size(med)) ///
          ysc(r(`yscl' `yscu')) xsc(r(0.5 3.5)) ///
          ylabel(`ylabl'(5)`ylablu', labsize(med)) ///
          xlabel(1 "Alter 20-39" 2 "Alter 40-59" 3 "Alter 60+" 4.6 "Gesamt",
labsize(med)) ///
          xtitle("", margin(0 0 0 2)) ///
      legend(cols(1) label(1 "Erwerbseinkommen") label(2 "Transfereinkommen")
label(3 "Andere Einkommen") ///
          label(7 "Gesamtänderung") order(1 2 3 7) rowgap(*0.8) ///
          bmargin(small) size(med) ring(0) position(11) symx(*0.8))
      graph display, xsize(`xs') ysize(`ys')
          graph export $graphslocation/diff_inc_AT.tif, replace
          graph export $graphslocation/dec_`cnt'_`sex'.png, replace

          browse AGEVAR DRYN1 D0pos D1pos D2pos D0neg D1neg D2neg
          order AGEVAR DRYN1 D0pos D1pos D2pos D0neg D1neg D2neg

  local sex = 1
  graph twoway bar DRYN0pos AGEVAR if SEX==`sex' & CNT2=="`cnt'", barw(`bw') col("0
29 74") lcol(`lc')  ///
      || bar DRYN1pos AGEVAR if SEX==`sex' & CNT2=="`cnt'", barw(`bw') col("48 122
133") lcol(`lc') ///
          || bar DRYN2pos AGEVAR if SEX==`sex' & CNT2=="`cnt'", barw(`bw') col("255
180 0") lcol(`lc') ///
          || bar DRYN3pos AGEVAR if SEX==`sex' & CNT2=="`cnt'", barw(`bw')
col("orange") lcol(`lc') ///
          || bar DRYN4pos AGEVAR if SEX==`sex' & CNT2=="`cnt'", barw(`bw') col("190
190 190") lcol(`lc') ///
      || bar DRYN0neg AGEVAR if SEX==`sex' & CNT2=="`cnt'", barw(`bw') col("0 29
74") lcol(`lc') ///
      || bar DRYN1neg AGEVAR if SEX==`sex' & CNT2=="`cnt'", barw(`bw') col("48 122
133") lcol(`lc') ///
          || bar DRYN2neg AGEVAR if SEX==`sex' & CNT2=="`cnt'", barw(`bw') col("255
180 0") lcol(`lc') ///
          || bar DRYN3neg AGEVAR if SEX==`sex' & CNT2=="`cnt'", barw(`bw')
col("orange") lcol(`lc') ///
          || bar DRYN4neg AGEVAR if SEX==`sex' & CNT2=="`cnt'", barw(`bw') col("190
190 190") lcol(`lc') ///
          || scatter DRYN1 AGEVAR if SEX==`sex' & CNT2=="`cnt'", col(black)  ///
          yline(0, lcol(gs5)) ///
          graphregion(margin(l-3 r-2 t+0 b-3) color(white)) ///
```

```
        plotregion(margin(tiny)) ///
    title("`cnt', men", size(med) margin(tiny)) ///
    ytitle("", size(medsmall)) ///
        ysc(r(`yscl' `yscu')) xsc(r(0.5 3.5)) ///
        ylabel(`ylabl'(5)`ylablu') ///
        xlabel(1 "20-39" 2 "40-59" 3 "60+" 4.6 "Total", labsize(medsmall)) ///
        xtitle("", margin(0 0 0 2)) ///
    legend(off)
    graph display, xsize(`xs') ysize(`ys')
        graph save $graphslocation/dec_`cnt'_`sex', replace
        graph export $graphslocation/dec_`cnt'_`sex'.png, replace

  local sex = 2
  graph twoway bar DRYN0pos AGEVAR if SEX==`sex' & CNT2=="`cnt'", barw(`bw') col("0
29 74") lcol(`lc')  ///
    || bar DRYN1pos AGEVAR if SEX==`sex' & CNT2=="`cnt'", barw(`bw') col("48 122
133") lcol(`lc') ///
        || bar DRYN2pos AGEVAR if SEX==`sex' & CNT2=="`cnt'", barw(`bw') col("255
180 0") lcol(`lc') ///
        || bar DRYN3pos AGEVAR if SEX==`sex' & CNT2=="`cnt'", barw(`bw')
col("orange") lcol(`lc') ///
        || bar DRYN4pos AGEVAR if SEX==`sex' & CNT2=="`cnt'", barw(`bw') col("190
190 190") lcol(`lc') ///
    || bar DRYN0neg AGEVAR if SEX==`sex' & CNT2=="`cnt'", barw(`bw') col("0 29
74") lcol(`lc') ///
    || bar DRYN1neg AGEVAR if SEX==`sex' & CNT2=="`cnt'", barw(`bw') col("48 122
133") lcol(`lc') ///
        || bar DRYN2neg AGEVAR if SEX==`sex' & CNT2=="`cnt'", barw(`bw') col("255
180 0") lcol(`lc') ///
        || bar DRYN3neg AGEVAR if SEX==`sex' & CNT2=="`cnt'", barw(`bw')
col("orange") lcol(`lc') ///
        || bar DRYN4neg AGEVAR if SEX==`sex' & CNT2=="`cnt'", barw(`bw') col("190
190 190") lcol(`lc') ///
        || scatter DRYN1 AGEVAR if SEX==`sex' & CNT2=="`cnt'", col(black)  ///
        yline(0, lcol(gs5)) ///
        graphregion(margin(l-3 r-2 t+0 b-3) color(white)) ///
        plotregion(margin(tiny)) ///
    title("`cnt', women", size(med) margin(tiny)) ///
    ytitle("", size(medsmall)) ///
        ysc(r(`yscl' `yscu')) xsc(r(0.5 3.5)) ///
        ylabel(`ylabl'(5)`ylablu') ///
        xlabel(1 "20-39" 2 "40-59" 3 "60+" 4.6 "Total", labsize(medsmall)) ///
        xtitle("", margin(0 0 0 2)) ///
    legend(off)
    graph display, xsize(`xs') ysize(`ys')
        graph save $graphslocation/dec_`cnt'_`sex', replace
        graph export $graphslocation/dec_`cnt'_`sex'.png, replace

       grc1leg $graphslocation/dec_AT_0.gph $graphslocation/dec_AT_1.gph
$graphslocation/dec_AT_2.gph, ///
```

```
        legendfrom($graphslocation/dec_AT_0.gph) cols(3) ///
        graphregion(margin(l-3 r-2 t+0 b-3) color(white)) ///
        plotregion(margin(tiny))


*******************************
* Estonia
*******************************

  local cnt "EE"
  local sex = 0
  local bw = 0.6
  local yscl = -25
  local yscu = 43
  local ylabl = -20
  local ylablu = 40

  graph twoway bar DRYN0pos AGEVAR if SEX==`sex' & CNT2=="`cnt'", barw(`bw') col("0
29 74") lcol(`lc')  ///
      || bar DRYN1pos AGEVAR if SEX==`sex' & CNT2=="`cnt'", barw(`bw') col("48 122
133") lcol(`lc') ///
          || bar DRYN2pos AGEVAR if SEX==`sex' & CNT2=="`cnt'", barw(`bw') col("255
180 0") lcol(`lc') ///
          || bar DRYN3pos AGEVAR if SEX==`sex' & CNT2=="`cnt'", barw(`bw')
col("orange") lcol(`lc') ///
          || bar DRYN4pos AGEVAR if SEX==`sex' & CNT2=="`cnt'", barw(`bw') col("190
190 190") lcol(`lc') ///
      || bar DRYN0neg AGEVAR if SEX==`sex' & CNT2=="`cnt'", barw(`bw') col("0 29
74") lcol(`lc') ///
      || bar DRYN1neg AGEVAR if SEX==`sex' & CNT2=="`cnt'", barw(`bw') col("48 122
133") lcol(`lc') ///
          || bar DRYN2neg AGEVAR if SEX==`sex' & CNT2=="`cnt'", barw(`bw') col("255
180 0") lcol(`lc') ///
          || bar DRYN3neg AGEVAR if SEX==`sex' & CNT2=="`cnt'", barw(`bw')
col("orange") lcol(`lc') ///
          || bar DRYN4neg AGEVAR if SEX==`sex' & CNT2=="`cnt'", barw(`bw') col("190
190 190") lcol(`lc') ///
          || scatter DRYN1 AGEVAR if SEX==`sex' & CNT2=="`cnt'", col(black)  ///
          yline(0, lcol(gs5)) ///
          graphregion(margin(l-3 r-2 t+0 b-3) color(white)) ///
          plotregion(margin(tiny)) ///
      title("`cnt', men and women", size(med) margin(tiny)) ///
      ytitle("Income change 2008 - 2017 in %", size(medsmall)) ///
          ysc(r(`yscl' `yscu')) xsc(r(0.5 3.5)) ///
          ylabel(`ylabl'(10)`ylablu') ///
          xlabel(1 "20-39" 2 "40-59" 3 "60+" 4.6 "Total", labsize(medsmall)) ///
          xtitle("", margin(0 0 0 2)) ///
      legend(cols(2) label(1 "Labour income: Employment") label(2 "Labour income:
Wages") label(3 "Benefits: share of receivers") ///
          label(4 "Benefits per beneficiary") label(5 "Other income") label(11
```

```
"Total change") order(1 2 3 4 5 11) rowgap(*0.8) ///
          bmargin(small) size(small) ring(1) position(11) symx(*0.5))
      graph display, xsize(`xs') ysize(`ys')
          graph save $graphslocation/dec_`cnt'_`sex', replace
          graph export $graphslocation/dec_`cnt'_`sex'.png, replace

  local sex = 1
  graph twoway bar DRYN0pos AGEVAR if SEX==`sex' & CNT2=="`cnt'", barw(`bw') col("0
29 74") lcol(`lc')  ///
      || bar DRYN1pos AGEVAR if SEX==`sex' & CNT2=="`cnt'", barw(`bw') col("48 122
133") lcol(`lc') ///
          || bar DRYN2pos AGEVAR if SEX==`sex' & CNT2=="`cnt'", barw(`bw') col("255
180 0") lcol(`lc') ///
          || bar DRYN3pos AGEVAR if SEX==`sex' & CNT2=="`cnt'", barw(`bw')
col("orange") lcol(`lc') ///
          || bar DRYN4pos AGEVAR if SEX==`sex' & CNT2=="`cnt'", barw(`bw') col("190
190 190") lcol(`lc') ///
      || bar DRYN0neg AGEVAR if SEX==`sex' & CNT2=="`cnt'", barw(`bw') col("0 29
74") lcol(`lc') ///
      || bar DRYN1neg AGEVAR if SEX==`sex' & CNT2=="`cnt'", barw(`bw') col("48 122
133") lcol(`lc') ///
          || bar DRYN2neg AGEVAR if SEX==`sex' & CNT2=="`cnt'", barw(`bw') col("255
180 0") lcol(`lc') ///
          || bar DRYN3neg AGEVAR if SEX==`sex' & CNT2=="`cnt'", barw(`bw')
col("orange") lcol(`lc') ///
          || bar DRYN4neg AGEVAR if SEX==`sex' & CNT2=="`cnt'", barw(`bw') col("190
190 190") lcol(`lc') ///
          || scatter DRYN1 AGEVAR if SEX==`sex' & CNT2=="`cnt'", col(black)  ///
          yline(0, lcol(gs5)) ///
          graphregion(margin(l-3 r-2 t+0 b-3) color(white)) ///
          plotregion(margin(tiny)) ///
      title("`cnt', men", size(med) margin(tiny)) ///
      ytitle("", size(medsmall)) ///
          ysc(r(`yscl' `yscu')) xsc(r(0.5 3.5)) ///
          ylabel(`ylabl'(10)`ylablu') ///
          xlabel(1 "20-39" 2 "40-59" 3 "60+" 4.6 "Total", labsize(medsmall)) ///
          xtitle("", margin(0 0 0 2)) ///
      legend(off)
      graph display, xsize(`xs') ysize(`ys')
          graph save $graphslocation/dec_`cnt'_`sex', replace
          graph export $graphslocation/dec_`cnt'_`sex'.png, replace

  local sex = 2
  graph twoway bar DRYN0pos AGEVAR if SEX==`sex' & CNT2=="`cnt'", barw(`bw') col("0
29 74") lcol(`lc')  ///
      || bar DRYN1pos AGEVAR if SEX==`sex' & CNT2=="`cnt'", barw(`bw') col("48 122
133") lcol(`lc') ///
          || bar DRYN2pos AGEVAR if SEX==`sex' & CNT2=="`cnt'", barw(`bw') col("255
180 0") lcol(`lc') ///
          || bar DRYN3pos AGEVAR if SEX==`sex' & CNT2=="`cnt'", barw(`bw')
```

```
col("orange") lcol(`lc') ///
          || bar DRYN4pos AGEVAR if SEX==`sex' & CNT2=="`cnt'", barw(`bw') col("190
190 190") lcol(`lc') ///
      || bar DRYN0neg AGEVAR if SEX==`sex' & CNT2=="`cnt'", barw(`bw') col("0 29
74") lcol(`lc') ///
      || bar DRYN1neg AGEVAR if SEX==`sex' & CNT2=="`cnt'", barw(`bw') col("48 122
133") lcol(`lc') ///
          || bar DRYN2neg AGEVAR if SEX==`sex' & CNT2=="`cnt'", barw(`bw') col("255
180 0") lcol(`lc') ///
          || bar DRYN3neg AGEVAR if SEX==`sex' & CNT2=="`cnt'", barw(`bw')
col("orange") lcol(`lc') ///
          || bar DRYN4neg AGEVAR if SEX==`sex' & CNT2=="`cnt'", barw(`bw') col("190
190 190") lcol(`lc') ///
          || scatter DRYN1 AGEVAR if SEX==`sex' & CNT2=="`cnt'", col(black)  ///
          yline(0, lcol(gs5)) ///
          graphregion(margin(l-3 r-2 t+0 b-3) color(white)) ///
          plotregion(margin(tiny)) ///
      title("`cnt', women", size(med) margin(tiny)) ///
      ytitle("", size(medsmall)) ///
          ysc(r(`yscl' `yscu')) xsc(r(0.5 3.5)) ///
          ylabel(`ylabl'(10)`ylablu') ///
          xlabel(1 "20-39" 2 "40-59" 3 "60+" 4.6 "Total", labsize(medsmall)) ///
          xtitle("", margin(0 0 0 2)) ///
      legend(off)
      graph display, xsize(`xs') ysize(`ys')
          graph save $graphslocation/dec_`cnt'_`sex', replace
          graph export $graphslocation/dec_`cnt'_`sex'.png, replace

        grc1leg $graphslocation/dec_`cnt'_0.gph $graphslocation/dec_`cnt'_1.gph
$graphslocation/dec_`cnt'_2.gph, ///
        legendfrom($graphslocation/dec_`cnt'_0.gph) cols(3) ///
        graphregion(margin(l-3 r-2 t+0 b-3) color(white)) ///
        plotregion(margin(tiny))

*******************************
* Greece
*******************************

  local cnt "EL"
  local sex = 0
  local bw = 0.6
  local yscl = -46
  local yscu = 5
  local ylabl = -45
  local ylablu = 5

  graph twoway bar DRYN0pos AGEVAR if SEX==`sex' & CNT2=="`cnt'", barw(`bw') col("0
29 74") lcol(`lc')  ///
      || bar DRYN1pos AGEVAR if SEX==`sex' & CNT2=="`cnt'", barw(`bw') col("48 122
133") lcol(`lc') ///
```

```
          || bar DRYN2pos AGEVAR if SEX==`sex' & CNT2=="`cnt'", barw(`bw') col("255
180 0") lcol(`lc') ///
          || bar DRYN3pos AGEVAR if SEX==`sex' & CNT2=="`cnt'", barw(`bw')
col("orange") lcol(`lc') ///
          || bar DRYN4pos AGEVAR if SEX==`sex' & CNT2=="`cnt'", barw(`bw') col("190
190 190") lcol(`lc') ///
      || bar DRYN0neg AGEVAR if SEX==`sex' & CNT2=="`cnt'", barw(`bw') col("0 29
74") lcol(`lc') ///
      || bar DRYN1neg AGEVAR if SEX==`sex' & CNT2=="`cnt'", barw(`bw') col("48 122
133") lcol(`lc') ///
          || bar DRYN2neg AGEVAR if SEX==`sex' & CNT2=="`cnt'", barw(`bw') col("255
180 0") lcol(`lc') ///
          || bar DRYN3neg AGEVAR if SEX==`sex' & CNT2=="`cnt'", barw(`bw')
col("orange") lcol(`lc') ///
          || bar DRYN4neg AGEVAR if SEX==`sex' & CNT2=="`cnt'", barw(`bw') col("190
190 190") lcol(`lc') ///
          || scatter DRYN1 AGEVAR if SEX==`sex' & CNT2=="`cnt'", col(black)  ///
          yline(0, lcol(gs5)) ///
          graphregion(margin(l-3 r-2 t+0 b-3) color(white)) ///
          plotregion(margin(tiny)) ///
      title("`cnt', men and women", size(med) margin(tiny)) ///
      ytitle("Income change 2008 - 2017 in %", size(medsmall)) ///
          ysc(r(`yscl' `yscu')) xsc(r(0.5 3.5)) ///
          ylabel(`ylabl'(10)`ylablu') ///
          xlabel(1 "20-39" 2 "40-59" 3 "60+" 4.6 "Total", labsize(medsmall)) ///

          xtitle("", margin(0 0 0 2)) ///
      legend(cols(2) label(1 "Employment rates (l)") label(2 "Income per employed
(yl)") label(3 "Share of beneficiaries (n)") ///
          label(4 "Benefits per beneficiary (yb)") label(5 "Other income (YO)")
label(11 "Total change") order(1 2 3 4 5 11) rowgap(*0.8) ///
          bmargin(small) size(small) ring(1) position(11) symx(*0.5))
      graph display, xsize(`xs') ysize(`ys')
          graph save $graphslocation/dec_`cnt'_`sex', replace
          graph export $graphslocation/dec_`cnt'_`sex'.png, replace

  local sex = 1
  graph twoway bar DRYN0pos AGEVAR if SEX==`sex' & CNT2=="`cnt'", barw(`bw') col("0
29 74") lcol(`lc')  ///
      || bar DRYN1pos AGEVAR if SEX==`sex' & CNT2=="`cnt'", barw(`bw') col("48 122
133") lcol(`lc') ///
          || bar DRYN2pos AGEVAR if SEX==`sex' & CNT2=="`cnt'", barw(`bw') col("255
180 0") lcol(`lc') ///
          || bar DRYN3pos AGEVAR if SEX==`sex' & CNT2=="`cnt'", barw(`bw')
col("orange") lcol(`lc') ///
          || bar DRYN4pos AGEVAR if SEX==`sex' & CNT2=="`cnt'", barw(`bw') col("190
190 190") lcol(`lc') ///
      || bar DRYN0neg AGEVAR if SEX==`sex' & CNT2=="`cnt'", barw(`bw') col("0 29
74") lcol(`lc') ///
      || bar DRYN1neg AGEVAR if SEX==`sex' & CNT2=="`cnt'", barw(`bw') col("48 122
```

```
133") lcol(`lc') ///
          || bar DRYN2neg AGEVAR if SEX==`sex' & CNT2=="`cnt'", barw(`bw') col("255
180 0") lcol(`lc') ///
          || bar DRYN3neg AGEVAR if SEX==`sex' & CNT2=="`cnt'", barw(`bw')
col("orange") lcol(`lc') ///
          || bar DRYN4neg AGEVAR if SEX==`sex' & CNT2=="`cnt'", barw(`bw') col("190
190 190") lcol(`lc') ///
          || scatter DRYN1 AGEVAR if SEX==`sex' & CNT2=="`cnt'", col(black)  ///
          yline(0, lcol(gs5)) ///
          graphregion(margin(l-3 r-2 t+0 b-3) color(white)) ///
          plotregion(margin(tiny)) ///
      title("`cnt', men", size(med) margin(tiny)) ///
      ytitle("", size(medsmall)) ///
          ysc(r(`yscl' `yscu')) xsc(r(0.5 3.5)) ///
          ylabel(`ylabl'(10)`ylablu') ///
          xlabel(1 "20-39" 2 "40-59" 3 "60+" 4.6 "Total", labsize(medsmall)) ///
          xtitle("", margin(0 0 0 2)) ///
      legend(off)
      graph display, xsize(`xs') ysize(`ys')
          graph save $graphslocation/dec_`cnt'_`sex', replace
          graph export $graphslocation/dec_`cnt'_`sex'.png, replace

  local sex = 2
  graph twoway bar DRYN0pos AGEVAR if SEX==`sex' & CNT2=="`cnt'", barw(`bw') col("0
29 74") lcol(`lc')  ///
      || bar DRYN1pos AGEVAR if SEX==`sex' & CNT2=="`cnt'", barw(`bw') col("48 122
133") lcol(`lc') ///
          || bar DRYN2pos AGEVAR if SEX==`sex' & CNT2=="`cnt'", barw(`bw') col("255
180 0") lcol(`lc') ///
          || bar DRYN3pos AGEVAR if SEX==`sex' & CNT2=="`cnt'", barw(`bw')
col("orange") lcol(`lc') ///
          || bar DRYN4pos AGEVAR if SEX==`sex' & CNT2=="`cnt'", barw(`bw') col("190
190 190") lcol(`lc') ///
      || bar DRYN0neg AGEVAR if SEX==`sex' & CNT2=="`cnt'", barw(`bw') col("0 29
74") lcol(`lc') ///
      || bar DRYN1neg AGEVAR if SEX==`sex' & CNT2=="`cnt'", barw(`bw') col("48 122
133") lcol(`lc') ///
          || bar DRYN2neg AGEVAR if SEX==`sex' & CNT2=="`cnt'", barw(`bw') col("255
180 0") lcol(`lc') ///
          || bar DRYN3neg AGEVAR if SEX==`sex' & CNT2=="`cnt'", barw(`bw')
col("orange") lcol(`lc') ///
          || bar DRYN4neg AGEVAR if SEX==`sex' & CNT2=="`cnt'", barw(`bw') col("190
190 190") lcol(`lc') ///
          || scatter DRYN1 AGEVAR if SEX==`sex' & CNT2=="`cnt'", col(black)  ///
          yline(0, lcol(gs5)) ///
          graphregion(margin(l-3 r-2 t+0 b-3) color(white)) ///
          plotregion(margin(tiny)) ///
      title("`cnt', women", size(med) margin(tiny)) ///
      ytitle("", size(medsmall)) ///
          ysc(r(`yscl' `yscu')) xsc(r(0.5 3.5)) ///
```

```
          ylabel(`ylabl'(10)`ylablu') ///
          xlabel(1 "20-39" 2 "40-59" 3 "60+" 4.6 "Total", labsize(medsmall)) ///

          xtitle("", margin(0 0 0 2)) ///
      legend(off)
      graph display, xsize(`xs') ysize(`ys')
          graph save $graphslocation/dec_`cnt'_`sex', replace
          graph export $graphslocation/dec_`cnt'_`sex'.png, replace

        grc1leg $graphslocation/dec_`cnt'_0.gph $graphslocation/dec_`cnt'_1.gph
$graphslocation/dec_`cnt'_2.gph, ///
        legendfrom($graphslocation/dec_`cnt'_0.gph) cols(3) ///
        graphregion(margin(l-3 r-2 t+0 b-3) color(white)) ///
        plotregion(margin(tiny))



*******************************
* Spain
*******************************

  local cnt "ES"
  local sex = 0
  local bw = 0.6
  local yscl = -25
  local yscu = 20
  local ylabl = -25
  local ylablu = 20

  graph twoway bar DRYN0pos AGEVAR if SEX==`sex' & CNT2=="`cnt'", barw(`bw') col("0
29 74") lcol(`lc')  ///
      || bar DRYN1pos AGEVAR if SEX==`sex' & CNT2=="`cnt'", barw(`bw') col("48 122
133") lcol(`lc') ///
          || bar DRYN2pos AGEVAR if SEX==`sex' & CNT2=="`cnt'", barw(`bw') col("255
180 0") lcol(`lc') ///
          || bar DRYN3pos AGEVAR if SEX==`sex' & CNT2=="`cnt'", barw(`bw')
col("orange") lcol(`lc') ///
          || bar DRYN4pos AGEVAR if SEX==`sex' & CNT2=="`cnt'", barw(`bw') col("190
190 190") lcol(`lc') ///
      || bar DRYN0neg AGEVAR if SEX==`sex' & CNT2=="`cnt'", barw(`bw') col("0 29
74") lcol(`lc') ///
      || bar DRYN1neg AGEVAR if SEX==`sex' & CNT2=="`cnt'", barw(`bw') col("48 122
133") lcol(`lc') ///
          || bar DRYN2neg AGEVAR if SEX==`sex' & CNT2=="`cnt'", barw(`bw') col("255
180 0") lcol(`lc') ///
          || bar DRYN3neg AGEVAR if SEX==`sex' & CNT2=="`cnt'", barw(`bw')
col("orange") lcol(`lc') ///
          || bar DRYN4neg AGEVAR if SEX==`sex' & CNT2=="`cnt'", barw(`bw') col("190
190 190") lcol(`lc') ///
          || scatter DRYN1 AGEVAR if SEX==`sex' & CNT2=="`cnt'", col(black)  ///
```

```
        yline(0, lcol(gs5)) ///
        graphregion(margin(l-3 r-2 t+0 b-3) color(white)) ///
        plotregion(margin(tiny)) ///
    title("`cnt', men and women", size(med) margin(tiny)) ///
    ytitle("Income change 2008 - 2017 in %", size(medsmall)) ///
        ysc(r(`yscl' `yscu')) xsc(r(0.5 3.5)) ///
        ylabel(`ylabl'(10)`ylablu') ///
        xlabel(1 "20-39" 2 "40-59" 3 "60+" 4.6 "Total", labsize(medsmall)) ///

        xtitle("", margin(0 0 0 2)) ///
    legend(cols(2) label(1 "Labour income: Employment") label(2 "Labour income: 
Wages") label(3 "Benefits: share of receivers") ///
        label(4 "Benefits per beneficiary") label(5 "Other income") label(11 
"Total change") order(1 2 3 4 5 11) rowgap(*0.8) ///
        bmargin(small) size(small) ring(1) position(11) symx(*0.5))
    graph display, xsize(`xs') ysize(`ys')
        graph save $graphslocation/dec_`cnt'_`sex', replace
        graph export $graphslocation/dec_`cnt'_`sex'.png, replace

  local sex = 1
  graph twoway bar DRYN0pos AGEVAR if SEX==`sex' & CNT2=="`cnt'", barw(`bw') col("0 
29 74") lcol(`lc')  ///
    || bar DRYN1pos AGEVAR if SEX==`sex' & CNT2=="`cnt'", barw(`bw') col("48 122 
133") lcol(`lc') ///
        || bar DRYN2pos AGEVAR if SEX==`sex' & CNT2=="`cnt'", barw(`bw') col("255 
180 0") lcol(`lc') ///
        || bar DRYN3pos AGEVAR if SEX==`sex' & CNT2=="`cnt'", barw(`bw') 
col("orange") lcol(`lc') ///
        || bar DRYN4pos AGEVAR if SEX==`sex' & CNT2=="`cnt'", barw(`bw') col("190 
190 190") lcol(`lc') ///
    || bar DRYN0neg AGEVAR if SEX==`sex' & CNT2=="`cnt'", barw(`bw') col("0 29 
74") lcol(`lc') ///
    || bar DRYN1neg AGEVAR if SEX==`sex' & CNT2=="`cnt'", barw(`bw') col("48 122 
133") lcol(`lc') ///
        || bar DRYN2neg AGEVAR if SEX==`sex' & CNT2=="`cnt'", barw(`bw') col("255 
180 0") lcol(`lc') ///
        || bar DRYN3neg AGEVAR if SEX==`sex' & CNT2=="`cnt'", barw(`bw') 
col("orange") lcol(`lc') ///
        || bar DRYN4neg AGEVAR if SEX==`sex' & CNT2=="`cnt'", barw(`bw') col("190 
190 190") lcol(`lc') ///
        || scatter DRYN1 AGEVAR if SEX==`sex' & CNT2=="`cnt'", col(black)  ///
        yline(0, lcol(gs5)) ///
        graphregion(margin(l-3 r-2 t+0 b-3) color(white)) ///
        plotregion(margin(tiny)) ///
    title("`cnt', men", size(med) margin(tiny)) ///
    ytitle("", size(medsmall)) ///
        ysc(r(`yscl' `yscu')) xsc(r(0.5 3.5)) ///
        ylabel(`ylabl'(10)`ylablu') ///
        xlabel(1 "20-39" 2 "40-59" 3 "60+" 4.6 "Total", labsize(medsmall)) ///
        xtitle("", margin(0 0 0 2)) ///
```

```
      legend(off)
       graph display, xsize(`xs') ysize(`ys')
          graph save $graphslocation/dec_`cnt'_`sex', replace
          graph export $graphslocation/dec_`cnt'_`sex'.png, replace

  local sex = 2
  graph twoway bar DRYN0pos AGEVAR if SEX==`sex' & CNT2=="`cnt'", barw(`bw') col("0 29 74") lcol(`lc')  ///
      || bar DRYN1pos AGEVAR if SEX==`sex' & CNT2=="`cnt'", barw(`bw') col("48 122 133") lcol(`lc') ///
          || bar DRYN2pos AGEVAR if SEX==`sex' & CNT2=="`cnt'", barw(`bw') col("255 180 0") lcol(`lc') ///
          || bar DRYN3pos AGEVAR if SEX==`sex' & CNT2=="`cnt'", barw(`bw') col("orange") lcol(`lc') ///
          || bar DRYN4pos AGEVAR if SEX==`sex' & CNT2=="`cnt'", barw(`bw') col("190 190 190") lcol(`lc') ///
      || bar DRYN0neg AGEVAR if SEX==`sex' & CNT2=="`cnt'", barw(`bw') col("0 29 74") lcol(`lc') ///
      || bar DRYN1neg AGEVAR if SEX==`sex' & CNT2=="`cnt'", barw(`bw') col("48 122 133") lcol(`lc') ///
          || bar DRYN2neg AGEVAR if SEX==`sex' & CNT2=="`cnt'", barw(`bw') col("255 180 0") lcol(`lc') ///
          || bar DRYN3neg AGEVAR if SEX==`sex' & CNT2=="`cnt'", barw(`bw') col("orange") lcol(`lc') ///
          || bar DRYN4neg AGEVAR if SEX==`sex' & CNT2=="`cnt'", barw(`bw') col("190 190 190") lcol(`lc') ///
          || scatter DRYN1 AGEVAR if SEX==`sex' & CNT2=="`cnt'", col(black)  ///
          yline(0, lcol(gs5)) ///
          graphregion(margin(l-3 r-2 t+0 b-3) color(white)) ///
          plotregion(margin(tiny)) ///
      title("`cnt', women", size(med) margin(tiny)) ///
      ytitle("", size(medsmall)) ///
          ysc(r(`yscl' `yscu')) xsc(r(0.5 3.5)) ///
          ylabel(`ylabl'(10)`ylablu') ///
          xlabel(1 "20-39" 2 "40-59" 3 "60+" 4.6 "Total", labsize(medsmall)) ///

          xtitle("", margin(0 0 0 2)) ///
      legend(off)
      graph display, xsize(`xs') ysize(`ys')
          graph save $graphslocation/dec_`cnt'_`sex', replace
          graph export $graphslocation/dec_`cnt'_`sex'.png, replace

        grc1leg $graphslocation/dec_`cnt'_0.gph $graphslocation/dec_`cnt'_1.gph $graphslocation/dec_`cnt'_2.gph, ///
        legendfrom($graphslocation/dec_`cnt'_0.gph) cols(3) ///
        graphregion(margin(l-3 r-2 t+0 b-3) color(white)) ///
        plotregion(margin(tiny))


*******************************
```

```
* France
******************************

  local cnt "FR"
  local sex = 0
  local bw = 0.6
  local yscl = -15
  local yscu = 23
  local ylabl = -15
  local ylablu = 20

  graph twoway bar DRYN0pos AGEVAR if SEX==`sex' & CNT2=="`cnt'", barw(`bw') col("0
29 74") lcol(`lc')  ///
      || bar DRYN1pos AGEVAR if SEX==`sex' & CNT2=="`cnt'", barw(`bw') col("48 122
133") lcol(`lc') ///
          || bar DRYN2pos AGEVAR if SEX==`sex' & CNT2=="`cnt'", barw(`bw') col("255
180 0") lcol(`lc') ///
          || bar DRYN3pos AGEVAR if SEX==`sex' & CNT2=="`cnt'", barw(`bw')
col("orange") lcol(`lc') ///
          || bar DRYN4pos AGEVAR if SEX==`sex' & CNT2=="`cnt'", barw(`bw') col("190
190 190") lcol(`lc') ///
      || bar DRYN0neg AGEVAR if SEX==`sex' & CNT2=="`cnt'", barw(`bw') col("0 29
74") lcol(`lc') ///
      || bar DRYN1neg AGEVAR if SEX==`sex' & CNT2=="`cnt'", barw(`bw') col("48 122
133") lcol(`lc') ///
          || bar DRYN2neg AGEVAR if SEX==`sex' & CNT2=="`cnt'", barw(`bw') col("255
180 0") lcol(`lc') ///
          || bar DRYN3neg AGEVAR if SEX==`sex' & CNT2=="`cnt'", barw(`bw')
col("orange") lcol(`lc') ///
          || bar DRYN4neg AGEVAR if SEX==`sex' & CNT2=="`cnt'", barw(`bw') col("190
190 190") lcol(`lc') ///
          || scatter DRYN1 AGEVAR if SEX==`sex' & CNT2=="`cnt'", col(black)  ///
          yline(0, lcol(gs5)) ///
          graphregion(margin(l-3 r-2 t+0 b-3) color(white)) ///
          plotregion(margin(tiny)) ///
      title("`cnt', men and women", size(med) margin(tiny)) ///
      ytitle("Income change 2008 - 2017 in %", size(medsmall)) ///
          ysc(r(`yscl' `yscu')) xsc(r(0.5 3.5)) ///
          ylabel(`ylabl'(5)`ylablu') ///
          xlabel(1 "20-39" 2 "40-59" 3 "60+" 4.6 "Total", labsize(medsmall)) ///

          xtitle("", margin(0 0 0 2)) ///
      legend(cols(2) label(1 "Labour income: Employment") label(2 "Labour income:
Wages") label(3 "Benefits: share of receivers") ///
          label(4 "Benefits per beneficiary") label(5 "Other income") label(11
"Total change") order(1 2 3 4 5 11) rowgap(*0.8) ///
          bmargin(small) size(small) ring(1) position(11) symx(*0.5))
      graph display, xsize(`xs') ysize(`ys')
          graph save $graphslocation/dec_`cnt'_`sex', replace
          graph export $graphslocation/dec_`cnt'_`sex'.png, replace
```

```
  local sex = 1
  graph twoway bar DRYN0pos AGEVAR if SEX==`sex' & CNT2=="`cnt'", barw(`bw') col("0
29 74") lcol(`lc')  ///
      || bar DRYN1pos AGEVAR if SEX==`sex' & CNT2=="`cnt'", barw(`bw') col("48 122
133") lcol(`lc') ///
          || bar DRYN2pos AGEVAR if SEX==`sex' & CNT2=="`cnt'", barw(`bw') col("255
180 0") lcol(`lc') ///
          || bar DRYN3pos AGEVAR if SEX==`sex' & CNT2=="`cnt'", barw(`bw')
col("orange") lcol(`lc') ///
          || bar DRYN4pos AGEVAR if SEX==`sex' & CNT2=="`cnt'", barw(`bw') col("190
190 190") lcol(`lc') ///
      || bar DRYN0neg AGEVAR if SEX==`sex' & CNT2=="`cnt'", barw(`bw') col("0 29
74") lcol(`lc') ///
      || bar DRYN1neg AGEVAR if SEX==`sex' & CNT2=="`cnt'", barw(`bw') col("48 122
133") lcol(`lc') ///
          || bar DRYN2neg AGEVAR if SEX==`sex' & CNT2=="`cnt'", barw(`bw') col("255
180 0") lcol(`lc') ///
          || bar DRYN3neg AGEVAR if SEX==`sex' & CNT2=="`cnt'", barw(`bw')
col("orange") lcol(`lc') ///
          || bar DRYN4neg AGEVAR if SEX==`sex' & CNT2=="`cnt'", barw(`bw') col("190
190 190") lcol(`lc') ///
          || scatter DRYN1 AGEVAR if SEX==`sex' & CNT2=="`cnt'", col(black)  ///
          yline(0, lcol(gs5)) ///
          graphregion(margin(l-3 r-2 t+0 b-3) color(white)) ///
          plotregion(margin(tiny)) ///
      title("`cnt', men", size(med) margin(tiny)) ///
      ytitle("", size(medsmall)) ///
          ysc(r(`yscl' `yscu')) xsc(r(0.5 3.5)) ///
          ylabel(`ylabl'(5)`ylablu') ///
          xlabel(1 "20-39" 2 "40-59" 3 "60+" 4.6 "Total", labsize(medsmall)) ///
          xtitle("", margin(0 0 0 2)) ///
      legend(off)
      graph display, xsize(`xs') ysize(`ys')
          graph save $graphslocation/dec_`cnt'_`sex', replace
          graph export $graphslocation/dec_`cnt'_`sex'.png, replace

  local sex = 2
  graph twoway bar DRYN0pos AGEVAR if SEX==`sex' & CNT2=="`cnt'", barw(`bw') col("0
29 74") lcol(`lc')  ///
      || bar DRYN1pos AGEVAR if SEX==`sex' & CNT2=="`cnt'", barw(`bw') col("48 122
133") lcol(`lc') ///
          || bar DRYN2pos AGEVAR if SEX==`sex' & CNT2=="`cnt'", barw(`bw') col("255
180 0") lcol(`lc') ///
          || bar DRYN3pos AGEVAR if SEX==`sex' & CNT2=="`cnt'", barw(`bw')
col("orange") lcol(`lc') ///
          || bar DRYN4pos AGEVAR if SEX==`sex' & CNT2=="`cnt'", barw(`bw') col("190
190 190") lcol(`lc') ///
      || bar DRYN0neg AGEVAR if SEX==`sex' & CNT2=="`cnt'", barw(`bw') col("0 29
74") lcol(`lc') ///
```

```
      || bar DRYN1neg AGEVAR if SEX==`sex' & CNT2=="`cnt'", barw(`bw') col("48 122
133") lcol(`lc') ///
          || bar DRYN2neg AGEVAR if SEX==`sex' & CNT2=="`cnt'", barw(`bw') col("255
180 0") lcol(`lc') ///
          || bar DRYN3neg AGEVAR if SEX==`sex' & CNT2=="`cnt'", barw(`bw')
col("orange") lcol(`lc') ///
          || bar DRYN4neg AGEVAR if SEX==`sex' & CNT2=="`cnt'", barw(`bw') col("190
190 190") lcol(`lc') ///
          || scatter DRYN1 AGEVAR if SEX==`sex' & CNT2=="`cnt'", col(black)  ///
          yline(0, lcol(gs5)) ///
          graphregion(margin(l-3 r-2 t+0 b-3) color(white)) ///
          plotregion(margin(tiny)) ///
      title("`cnt', women", size(med) margin(tiny)) ///
      ytitle("", size(medsmall)) ///
          ysc(r(`yscl' `yscu')) xsc(r(0.5 3.5)) ///
          ylabel(`ylabl'(5)`ylablu') ///
          xlabel(1 "20-39" 2 "40-59" 3 "60+" 4.6 "Total", labsize(medsmall)) ///

          xtitle("", margin(0 0 0 2)) ///
      legend(off)
      graph display, xsize(`xs') ysize(`ys')
          graph save $graphslocation/dec_`cnt'_`sex', replace
          graph export $graphslocation/dec_`cnt'_`sex'.png, replace

        grc1leg $graphslocation/dec_`cnt'_0.gph $graphslocation/dec_`cnt'_1.gph
$graphslocation/dec_`cnt'_2.gph, ///
        legendfrom($graphslocation/dec_`cnt'_0.gph) cols(3) ///
        graphregion(margin(l-3 r-2 t+0 b-3) color(white)) ///
        plotregion(margin(tiny))


*******************************
* Italy
*******************************

  local cnt "IT"
  local sex = 0
  local bw = 0.6
  local yscl = -22
  local yscu = 12
  local ylabl = -20
  local ylablu = 10

  graph twoway bar DRYN0pos AGEVAR if SEX==`sex' & CNT2=="`cnt'", barw(`bw') col("0
29 74") lcol(`lc')  ///
      || bar DRYN1pos AGEVAR if SEX==`sex' & CNT2=="`cnt'", barw(`bw') col("48 122
133") lcol(`lc') ///
          || bar DRYN2pos AGEVAR if SEX==`sex' & CNT2=="`cnt'", barw(`bw') col("255
180 0") lcol(`lc') ///
          || bar DRYN3pos AGEVAR if SEX==`sex' & CNT2=="`cnt'", barw(`bw')
```

```
col("orange") lcol(`lc') ///
          || bar DRYN4pos AGEVAR if SEX==`sex' & CNT2=="`cnt'", barw(`bw') col("190
190 190") lcol(`lc') ///
      || bar DRYN0neg AGEVAR if SEX==`sex' & CNT2=="`cnt'", barw(`bw') col("0 29
74") lcol(`lc') ///
      || bar DRYN1neg AGEVAR if SEX==`sex' & CNT2=="`cnt'", barw(`bw') col("48 122
133") lcol(`lc') ///
          || bar DRYN2neg AGEVAR if SEX==`sex' & CNT2=="`cnt'", barw(`bw') col("255
180 0") lcol(`lc') ///
          || bar DRYN3neg AGEVAR if SEX==`sex' & CNT2=="`cnt'", barw(`bw')
col("orange") lcol(`lc') ///
          || bar DRYN4neg AGEVAR if SEX==`sex' & CNT2=="`cnt'", barw(`bw') col("190
190 190") lcol(`lc') ///
          || scatter DRYN1 AGEVAR if SEX==`sex' & CNT2=="`cnt'", col(black)  ///
          yline(0, lcol(gs5)) ///
          graphregion(margin(l-3 r-2 t+0 b-3) color(white)) ///
          plotregion(margin(tiny)) ///
      title("`cnt', men and women", size(med) margin(tiny)) ///
      ytitle("Income change 2008 - 2017 in %", size(medsmall)) ///
          ysc(r(`yscl' `yscu')) xsc(r(0.5 3.5)) ///
          ylabel(`ylabl'(10)`ylablu') ///
          xlabel(1 "20-39" 2 "40-59" 3 "60+" 4.6 "Total", labsize(medsmall)) ///

          xtitle("", margin(0 0 0 2)) ///
      legend(cols(2) label(1 "Employment rates (l)") label(2 "Income per employed
(yl)") label(3 "Share of beneficiaries (n)") ///
          label(4 "Benefits per beneficiary (yb)") label(5 "Other income (YO)")
label(11 "Total change") order(1 2 3 4 5 11) rowgap(*0.8) ///
          bmargin(small) size(small) ring(1) position(11) symx(*0.5))
       graph display, xsize(`xs') ysize(`ys')
          graph save $graphslocation/dec_`cnt'_`sex', replace
          graph export $graphslocation/dec_`cnt'_`sex'.png, replace

  local sex = 1
  graph twoway bar DRYN0pos AGEVAR if SEX==`sex' & CNT2=="`cnt'", barw(`bw') col("0
29 74") lcol(`lc')  ///
      || bar DRYN1pos AGEVAR if SEX==`sex' & CNT2=="`cnt'", barw(`bw') col("48 122
133") lcol(`lc') ///
          || bar DRYN2pos AGEVAR if SEX==`sex' & CNT2=="`cnt'", barw(`bw') col("255
180 0") lcol(`lc') ///
          || bar DRYN3pos AGEVAR if SEX==`sex' & CNT2=="`cnt'", barw(`bw')
col("orange") lcol(`lc') ///
          || bar DRYN4pos AGEVAR if SEX==`sex' & CNT2=="`cnt'", barw(`bw') col("190
190 190") lcol(`lc') ///
      || bar DRYN0neg AGEVAR if SEX==`sex' & CNT2=="`cnt'", barw(`bw') col("0 29
74") lcol(`lc') ///
      || bar DRYN1neg AGEVAR if SEX==`sex' & CNT2=="`cnt'", barw(`bw') col("48 122
133") lcol(`lc') ///
          || bar DRYN2neg AGEVAR if SEX==`sex' & CNT2=="`cnt'", barw(`bw') col("255
180 0") lcol(`lc') ///
```

```
          || bar DRYN3neg AGEVAR if SEX==`sex' & CNT2=="`cnt'", barw(`bw')
col("orange") lcol(`lc') ///
          || bar DRYN4neg AGEVAR if SEX==`sex' & CNT2=="`cnt'", barw(`bw') col("190
190 190") lcol(`lc') ///
          || scatter DRYN1 AGEVAR if SEX==`sex' & CNT2=="`cnt'", col(black)  ///
          yline(0, lcol(gs5)) ///
          graphregion(margin(l-3 r-2 t+0 b-3) color(white)) ///
          plotregion(margin(tiny)) ///
      title("`cnt', men", size(med) margin(tiny)) ///
      ytitle("", size(medsmall)) ///
          ysc(r(`yscl' `yscu')) xsc(r(0.5 3.5)) ///
          ylabel(`ylabl'(10)`ylablu') ///
          xlabel(1 "20-39" 2 "40-59" 3 "60+" 4.6 "Total", labsize(medsmall)) ///
          xtitle("", margin(0 0 0 2)) ///
      legend(off)
      graph display, xsize(`xs') ysize(`ys')
          graph save $graphslocation/dec_`cnt'_`sex', replace
          graph export $graphslocation/dec_`cnt'_`sex'.png, replace

  local sex = 2
  graph twoway bar DRYN0pos AGEVAR if SEX==`sex' & CNT2=="`cnt'", barw(`bw') col("0
29 74") lcol(`lc')  ///
      || bar DRYN1pos AGEVAR if SEX==`sex' & CNT2=="`cnt'", barw(`bw') col("48 122
133") lcol(`lc') ///
          || bar DRYN2pos AGEVAR if SEX==`sex' & CNT2=="`cnt'", barw(`bw') col("255
180 0") lcol(`lc') ///
          || bar DRYN3pos AGEVAR if SEX==`sex' & CNT2=="`cnt'", barw(`bw')
col("orange") lcol(`lc') ///
          || bar DRYN4pos AGEVAR if SEX==`sex' & CNT2=="`cnt'", barw(`bw') col("190
190 190") lcol(`lc') ///
      || bar DRYN0neg AGEVAR if SEX==`sex' & CNT2=="`cnt'", barw(`bw') col("0 29
74") lcol(`lc') ///
      || bar DRYN1neg AGEVAR if SEX==`sex' & CNT2=="`cnt'", barw(`bw') col("48 122
133") lcol(`lc') ///
          || bar DRYN2neg AGEVAR if SEX==`sex' & CNT2=="`cnt'", barw(`bw') col("255
180 0") lcol(`lc') ///
          || bar DRYN3neg AGEVAR if SEX==`sex' & CNT2=="`cnt'", barw(`bw')
col("orange") lcol(`lc') ///
          || bar DRYN4neg AGEVAR if SEX==`sex' & CNT2=="`cnt'", barw(`bw') col("190
190 190") lcol(`lc') ///
          || scatter DRYN1 AGEVAR if SEX==`sex' & CNT2=="`cnt'", col(black)  ///
          yline(0, lcol(gs5)) ///
          graphregion(margin(l-3 r-2 t+0 b-3) color(white)) ///
          plotregion(margin(tiny)) ///
      title("`cnt', women", size(med) margin(tiny)) ///
      ytitle("", size(medsmall)) ///
          ysc(r(`yscl' `yscu')) xsc(r(0.5 3.5)) ///
          ylabel(`ylabl'(10)`ylablu') ///
          xlabel(1 "20-39" 2 "40-59" 3 "60+" 4.6 "Total", labsize(medsmall)) ///
```

```
            xtitle("", margin(0 0 0 2)) ///
        legend(off)
        graph display, xsize(`xs') ysize(`ys')
            graph save $graphslocation/dec_`cnt'_`sex', replace
            graph export $graphslocation/dec_`cnt'_`sex'.png, replace

          grc1leg $graphslocation/dec_`cnt'_0.gph $graphslocation/dec_`cnt'_1.gph
$graphslocation/dec_`cnt'_2.gph, ///
          legendfrom($graphslocation/dec_`cnt'_0.gph) cols(3) ///
          graphregion(margin(l-3 r-2 t+0 b-3) color(white)) ///
          plotregion(margin(tiny))


*******************************
* Poland
*******************************

  local cnt "PL"
  local sex = 0
  local bw = 0.6
  local yscl = -15
  local yscu = 15
  local ylabl = -15
  local ylablu = 15

  graph twoway bar DRYN0pos AGEVAR if SEX==`sex' & CNT2=="`cnt'", barw(`bw') col("0
29 74") lcol(`lc')  ///
      || bar DRYN1pos AGEVAR if SEX==`sex' & CNT2=="`cnt'", barw(`bw') col("48 122
133") lcol(`lc') ///
          || bar DRYN2pos AGEVAR if SEX==`sex' & CNT2=="`cnt'", barw(`bw') col("255
180 0") lcol(`lc') ///
          || bar DRYN3pos AGEVAR if SEX==`sex' & CNT2=="`cnt'", barw(`bw')
col("orange") lcol(`lc') ///
          || bar DRYN4pos AGEVAR if SEX==`sex' & CNT2=="`cnt'", barw(`bw') col("190
190 190") lcol(`lc') ///
      || bar DRYN0neg AGEVAR if SEX==`sex' & CNT2=="`cnt'", barw(`bw') col("0 29
74") lcol(`lc') ///
      || bar DRYN1neg AGEVAR if SEX==`sex' & CNT2=="`cnt'", barw(`bw') col("48 122
133") lcol(`lc') ///
          || bar DRYN2neg AGEVAR if SEX==`sex' & CNT2=="`cnt'", barw(`bw') col("255
180 0") lcol(`lc') ///
          || bar DRYN3neg AGEVAR if SEX==`sex' & CNT2=="`cnt'", barw(`bw')
col("orange") lcol(`lc') ///
          || bar DRYN4neg AGEVAR if SEX==`sex' & CNT2=="`cnt'", barw(`bw') col("190
190 190") lcol(`lc') ///
          || scatter DRYN1 AGEVAR if SEX==`sex' & CNT2=="`cnt'", col(black)  ///
          yline(0, lcol(gs5)) ///
          graphregion(margin(l-3 r-2 t+0 b-3) color(white)) ///
          plotregion(margin(tiny)) ///
      title("`cnt', men and women", size(med) margin(tiny)) ///
```

```
      ytitle("Income change 2008 - 2017 in %", size(medsmall)) ///
          ysc(r(`yscl' `yscu')) xsc(r(0.5 3.5)) ///
          ylabel(`ylabl'(5)`ylablu') ///
          xlabel(1 "20-39" 2 "40-59" 3 "60+" 4.6 "Total", labsize(medsmall)) ///

          xtitle("", margin(0 0 0 2)) ///
      legend(cols(2) label(1 "Labour income: Employment") label(2 "Labour income:
Wages") label(3 "Benefits: share of receivers") ///
          label(4 "Benefits per beneficiary") label(5 "Other income") label(11
"Total change") order(1 2 3 4 5 11) rowgap(*0.8) ///
          bmargin(small) size(small) ring(1) position(11) symx(*0.5))
      graph display, xsize(`xs') ysize(`ys')
          graph save $graphslocation/dec_`cnt'_`sex', replace
          graph export $graphslocation/dec_`cnt'_`sex'.png, replace

  local sex = 1
  graph twoway bar DRYN0pos AGEVAR if SEX==`sex' & CNT2=="`cnt'", barw(`bw') col("0
29 74") lcol(`lc')  ///
      || bar DRYN1pos AGEVAR if SEX==`sex' & CNT2=="`cnt'", barw(`bw') col("48 122
133") lcol(`lc') ///
          || bar DRYN2pos AGEVAR if SEX==`sex' & CNT2=="`cnt'", barw(`bw') col("255
180 0") lcol(`lc') ///
          || bar DRYN3pos AGEVAR if SEX==`sex' & CNT2=="`cnt'", barw(`bw')
col("orange") lcol(`lc') ///
          || bar DRYN4pos AGEVAR if SEX==`sex' & CNT2=="`cnt'", barw(`bw') col("190
190 190") lcol(`lc') ///
      || bar DRYN0neg AGEVAR if SEX==`sex' & CNT2=="`cnt'", barw(`bw') col("0 29
74") lcol(`lc') ///
      || bar DRYN1neg AGEVAR if SEX==`sex' & CNT2=="`cnt'", barw(`bw') col("48 122
133") lcol(`lc') ///
          || bar DRYN2neg AGEVAR if SEX==`sex' & CNT2=="`cnt'", barw(`bw') col("255
180 0") lcol(`lc') ///
          || bar DRYN3neg AGEVAR if SEX==`sex' & CNT2=="`cnt'", barw(`bw')
col("orange") lcol(`lc') ///
          || bar DRYN4neg AGEVAR if SEX==`sex' & CNT2=="`cnt'", barw(`bw') col("190
190 190") lcol(`lc') ///
          || scatter DRYN1 AGEVAR if SEX==`sex' & CNT2=="`cnt'", col(black)  ///
          yline(0, lcol(gs5)) ///
          graphregion(margin(l-3 r-2 t+0 b-3) color(white)) ///
          plotregion(margin(tiny)) ///
      title("`cnt', men", size(med) margin(tiny)) ///
      ytitle("", size(medsmall)) ///
          ysc(r(`yscl' `yscu')) xsc(r(0.5 3.5)) ///
          ylabel(`ylabl'(5)`ylablu') ///
          xlabel(1 "20-39" 2 "40-59" 3 "60+" 4.6 "Total", labsize(medsmall)) ///
          xtitle("", margin(0 0 0 2)) ///
      legend(off)
      graph display, xsize(`xs') ysize(`ys')
          graph save $graphslocation/dec_`cnt'_`sex', replace
          graph export $graphslocation/dec_`cnt'_`sex'.png, replace
```

```
  local sex = 2
  graph twoway bar DRYN0pos AGEVAR if SEX==`sex' & CNT2=="`cnt'", barw(`bw') col("0
29 74") lcol(`lc')  ///
      || bar DRYN1pos AGEVAR if SEX==`sex' & CNT2=="`cnt'", barw(`bw') col("48 122
133") lcol(`lc') ///
          || bar DRYN2pos AGEVAR if SEX==`sex' & CNT2=="`cnt'", barw(`bw') col("255
180 0") lcol(`lc') ///
          || bar DRYN3pos AGEVAR if SEX==`sex' & CNT2=="`cnt'", barw(`bw')
col("orange") lcol(`lc') ///
          || bar DRYN4pos AGEVAR if SEX==`sex' & CNT2=="`cnt'", barw(`bw') col("190
190 190") lcol(`lc') ///
      || bar DRYN0neg AGEVAR if SEX==`sex' & CNT2=="`cnt'", barw(`bw') col("0 29
74") lcol(`lc') ///
      || bar DRYN1neg AGEVAR if SEX==`sex' & CNT2=="`cnt'", barw(`bw') col("48 122
133") lcol(`lc') ///
          || bar DRYN2neg AGEVAR if SEX==`sex' & CNT2=="`cnt'", barw(`bw') col("255
180 0") lcol(`lc') ///
          || bar DRYN3neg AGEVAR if SEX==`sex' & CNT2=="`cnt'", barw(`bw')
col("orange") lcol(`lc') ///
          || bar DRYN4neg AGEVAR if SEX==`sex' & CNT2=="`cnt'", barw(`bw') col("190
190 190") lcol(`lc') ///
          || scatter DRYN1 AGEVAR if SEX==`sex' & CNT2=="`cnt'", col(black)  ///
          yline(0, lcol(gs5)) ///
          graphregion(margin(l-3 r-2 t+0 b-3) color(white)) ///
          plotregion(margin(tiny)) ///
      title("`cnt', women", size(med) margin(tiny)) ///
      ytitle("", size(medsmall)) ///
          ysc(r(`yscl' `yscu')) xsc(r(0.5 3.5)) ///
          ylabel(`ylabl'(5)`ylablu') ///
          xlabel(1 "20-39" 2 "40-59" 3 "60+" 4.6 "Total", labsize(medsmall)) ///

          xtitle("", margin(0 0 0 2)) ///
      legend(off)
      graph display, xsize(`xs') ysize(`ys')
          graph save $graphslocation/dec_`cnt'_`sex', replace
          graph export $graphslocation/dec_`cnt'_`sex'.png, replace

        grc1leg $graphslocation/dec_`cnt'_0.gph $graphslocation/dec_`cnt'_1.gph
$graphslocation/dec_`cnt'_2.gph, ///
        legendfrom($graphslocation/dec_`cnt'_0.gph) cols(3) ///
        graphregion(margin(l-3 r-2 t+0 b-3) color(white)) ///
        plotregion(margin(tiny))

*******************************
* Sweden
*******************************

  local cnt "SE"
  local sex = 0
```

```
  local bw = 0.6
  local yscl = -8
  local yscu = 24
  local ylabl = -5
  local ylablu = 20

  graph twoway bar DRYN0pos AGEVAR if SEX==`sex' & CNT2=="`cnt'", barw(`bw') col("0
29 74") lcol(`lc')  ///
      || bar DRYN1pos AGEVAR if SEX==`sex' & CNT2=="`cnt'", barw(`bw') col("48 122
133") lcol(`lc') ///
          || bar DRYN2pos AGEVAR if SEX==`sex' & CNT2=="`cnt'", barw(`bw') col("255
180 0") lcol(`lc') ///
          || bar DRYN3pos AGEVAR if SEX==`sex' & CNT2=="`cnt'", barw(`bw')
col("orange") lcol(`lc') ///
          || bar DRYN4pos AGEVAR if SEX==`sex' & CNT2=="`cnt'", barw(`bw') col("190
190 190") lcol(`lc') ///
      || bar DRYN0neg AGEVAR if SEX==`sex' & CNT2=="`cnt'", barw(`bw') col("0 29
74") lcol(`lc') ///
      || bar DRYN1neg AGEVAR if SEX==`sex' & CNT2=="`cnt'", barw(`bw') col("48 122
133") lcol(`lc') ///
          || bar DRYN2neg AGEVAR if SEX==`sex' & CNT2=="`cnt'", barw(`bw') col("255
180 0") lcol(`lc') ///
          || bar DRYN3neg AGEVAR if SEX==`sex' & CNT2=="`cnt'", barw(`bw')
col("orange") lcol(`lc') ///
          || bar DRYN4neg AGEVAR if SEX==`sex' & CNT2=="`cnt'", barw(`bw') col("190
190 190") lcol(`lc') ///
          || scatter DRYN1 AGEVAR if SEX==`sex' & CNT2=="`cnt'", col(black)  ///
          yline(0, lcol(gs5)) ///
          graphregion(margin(l-3 r-2 t+0 b-3) color(white)) ///
          plotregion(margin(tiny)) ///
      title("`cnt', men and women", size(med) margin(tiny)) ///
      ytitle("Income change 2008 - 2017 in %", size(medsmall)) ///
          ysc(r(`yscl' `yscu')) xsc(r(0.5 3.5)) ///
          ylabel(`ylabl'(5)`ylablu') ///
          xlabel(1 "20-39" 2 "40-59" 3 "60+" 4.6 "Total", labsize(medsmall)) ///

          xtitle("", margin(0 0 0 2)) ///
      legend(cols(2) label(1 "Labour income: Employment") label(2 "Labour income:
Wages") label(3 "Benefits: share of receivers") ///
          label(4 "Benefits per beneficiary") label(5 "Other income") label(11
"Total change") order(1 2 3 4 5 11) rowgap(*0.8) ///
          bmargin(small) size(small) ring(1) position(11) symx(*0.5))
      graph display, xsize(`xs') ysize(`ys')
          graph save $graphslocation/dec_`cnt'_`sex', replace
          graph export $graphslocation/dec_`cnt'_`sex'.png, replace

  local sex = 1
  graph twoway bar DRYN0pos AGEVAR if SEX==`sex' & CNT2=="`cnt'", barw(`bw') col("0
29 74") lcol(`lc')  ///
      || bar DRYN1pos AGEVAR if SEX==`sex' & CNT2=="`cnt'", barw(`bw') col("48 122
```

```
133") lcol(`lc') ///
          || bar DRYN2pos AGEVAR if SEX==`sex' & CNT2=="`cnt'", barw(`bw') col("255 
180 0") lcol(`lc') ///
          || bar DRYN3pos AGEVAR if SEX==`sex' & CNT2=="`cnt'", barw(`bw') 
col("orange") lcol(`lc') ///
          || bar DRYN4pos AGEVAR if SEX==`sex' & CNT2=="`cnt'", barw(`bw') col("190 
190 190") lcol(`lc') ///
      || bar DRYN0neg AGEVAR if SEX==`sex' & CNT2=="`cnt'", barw(`bw') col("0 29 
74") lcol(`lc') ///
      || bar DRYN1neg AGEVAR if SEX==`sex' & CNT2=="`cnt'", barw(`bw') col("48 122 
133") lcol(`lc') ///
          || bar DRYN2neg AGEVAR if SEX==`sex' & CNT2=="`cnt'", barw(`bw') col("255 
180 0") lcol(`lc') ///
          || bar DRYN3neg AGEVAR if SEX==`sex' & CNT2=="`cnt'", barw(`bw') 
col("orange") lcol(`lc') ///
          || bar DRYN4neg AGEVAR if SEX==`sex' & CNT2=="`cnt'", barw(`bw') col("190 
190 190") lcol(`lc') ///
          || scatter DRYN1 AGEVAR if SEX==`sex' & CNT2=="`cnt'", col(black)  ///
          yline(0, lcol(gs5)) ///
          graphregion(margin(l-3 r-2 t+0 b-3) color(white)) ///
          plotregion(margin(tiny)) ///
      title("`cnt', men", size(med) margin(tiny)) ///
      ytitle("", size(medsmall)) ///
          ysc(r(`yscl' `yscu')) xsc(r(0.5 3.5)) ///
          ylabel(`ylabl'(5)`ylablu') ///
          xlabel(1 "20-39" 2 "40-59" 3 "60+" 4.6 "Total", labsize(medsmall)) ///
          xtitle("", margin(0 0 0 2)) ///
      legend(off)
      graph display, xsize(`xs') ysize(`ys')
          graph save $graphslocation/dec_`cnt'_`sex', replace
          graph export $graphslocation/dec_`cnt'_`sex'.png, replace

  local sex = 2
  graph twoway bar DRYN0pos AGEVAR if SEX==`sex' & CNT2=="`cnt'", barw(`bw') col("0 
29 74") lcol(`lc')  ///
      || bar DRYN1pos AGEVAR if SEX==`sex' & CNT2=="`cnt'", barw(`bw') col("48 122 
133") lcol(`lc') ///
          || bar DRYN2pos AGEVAR if SEX==`sex' & CNT2=="`cnt'", barw(`bw') col("255 
180 0") lcol(`lc') ///
          || bar DRYN3pos AGEVAR if SEX==`sex' & CNT2=="`cnt'", barw(`bw') 
col("orange") lcol(`lc') ///
          || bar DRYN4pos AGEVAR if SEX==`sex' & CNT2=="`cnt'", barw(`bw') col("190 
190 190") lcol(`lc') ///
      || bar DRYN0neg AGEVAR if SEX==`sex' & CNT2=="`cnt'", barw(`bw') col("0 29 
74") lcol(`lc') ///
      || bar DRYN1neg AGEVAR if SEX==`sex' & CNT2=="`cnt'", barw(`bw') col("48 122 
133") lcol(`lc') ///
          || bar DRYN2neg AGEVAR if SEX==`sex' & CNT2=="`cnt'", barw(`bw') col("255 
180 0") lcol(`lc') ///
          || bar DRYN3neg AGEVAR if SEX==`sex' & CNT2=="`cnt'", barw(`bw') 
```

```
col("orange") lcol(`lc') ///
          || bar DRYN4neg AGEVAR if SEX==`sex' & CNT2=="`cnt'", barw(`bw') col("190
190 190") lcol(`lc') ///
          || scatter DRYN1 AGEVAR if SEX==`sex' & CNT2=="`cnt'", col(black)  ///
          yline(0, lcol(gs5)) ///
          graphregion(margin(l-3 r-2 t+0 b-3) color(white)) ///
          plotregion(margin(tiny)) ///
      title("`cnt', women", size(med) margin(tiny)) ///
      ytitle("", size(medsmall)) ///
          ysc(r(`yscl' `yscu')) xsc(r(0.5 3.5)) ///
          ylabel(`ylabl'(5)`ylablu') ///
          xlabel(1 "20-39" 2 "40-59" 3 "60+" 4.6 "Total", labsize(medsmall)) ///
          xtitle("", margin(0 0 0 2)) ///
      legend(off)
      graph display, xsize(`xs') ysize(`ys')
          graph save $graphslocation/dec_`cnt'_`sex', replace
          graph export $graphslocation/dec_`cnt'_`sex'.png, replace

        grc1leg $graphslocation/dec_`cnt'_0.gph $graphslocation/dec_`cnt'_1.gph
$graphslocation/dec_`cnt'_2.gph, ///
        legendfrom($graphslocation/dec_`cnt'_0.gph) cols(3) ///
        graphregion(margin(l-3 r-2 t+0 b-3) color(white)) ///
        plotregion(margin(tiny))


*******************************
* Slovenia
*******************************

  local cnt "SI"
  local sex = 0
  local bw = 0.6
  local yscl = -6
  local yscu = 15
  local ylabl = -5
  local ylablu = 15

  graph twoway bar DRYN0pos AGEVAR if SEX==`sex' & CNT2=="`cnt'", barw(`bw') col("0
29 74") lcol(`lc')  ///
      || bar DRYN1pos AGEVAR if SEX==`sex' & CNT2=="`cnt'", barw(`bw') col("48 122
133") lcol(`lc') ///
          || bar DRYN2pos AGEVAR if SEX==`sex' & CNT2=="`cnt'", barw(`bw') col("255
180 0") lcol(`lc') ///
          || bar DRYN3pos AGEVAR if SEX==`sex' & CNT2=="`cnt'", barw(`bw')
col("orange") lcol(`lc') ///
          || bar DRYN4pos AGEVAR if SEX==`sex' & CNT2=="`cnt'", barw(`bw') col("190
190 190") lcol(`lc') ///
      || bar DRYN0neg AGEVAR if SEX==`sex' & CNT2=="`cnt'", barw(`bw') col("0 29
74") lcol(`lc') ///
      || bar DRYN1neg AGEVAR if SEX==`sex' & CNT2=="`cnt'", barw(`bw') col("48 122
```

```
133") lcol(`lc') ///
          || bar DRYN2neg AGEVAR if SEX==`sex' & CNT2=="`cnt'", barw(`bw') col("255
180 0") lcol(`lc') ///
          || bar DRYN3neg AGEVAR if SEX==`sex' & CNT2=="`cnt'", barw(`bw')
col("orange") lcol(`lc') ///
          || bar DRYN4neg AGEVAR if SEX==`sex' & CNT2=="`cnt'", barw(`bw') col("190
190 190") lcol(`lc') ///
          || scatter DRYN1 AGEVAR if SEX==`sex' & CNT2=="`cnt'", col(black)  ///
          yline(0, lcol(gs5)) ///
          graphregion(margin(l-3 r-2 t+0 b-3) color(white)) ///
          plotregion(margin(tiny)) ///
      title("`cnt', men and women", size(med) margin(tiny)) ///
      ytitle("Income change 2008 - 2017 in %", size(medsmall)) ///
          ysc(r(`yscl' `yscu')) xsc(r(0.5 3.5)) ///
          ylabel(`ylabl'(5)`ylablu') ///
          xlabel(1 "20-39" 2 "40-59" 3 "60+" 4.6 "Total", labsize(medsmall)) ///

          xtitle("", margin(0 0 0 2)) ///
      legend(cols(2) label(1 "Employment rates (l)") label(2 "Income per employed
(yl)") label(3 "Share of beneficiaries (n)") ///
          label(4 "Benefits per beneficiary (yb)") label(5 "Other income (YO)")
label(11 "Total change") order(1 2 3 4 5 11) rowgap(*0.8) ///
          bmargin(small) size(small) ring(1) position(11) symx(*0.5))
      graph display, xsize(`xs') ysize(`ys')
          graph save $graphslocation/dec_`cnt'_`sex', replace
          graph export $graphslocation/dec_`cnt'_`sex'.png, replace

  local sex = 1
  graph twoway bar DRYN0pos AGEVAR if SEX==`sex' & CNT2=="`cnt'", barw(`bw') col("0
29 74") lcol(`lc')  ///
      || bar DRYN1pos AGEVAR if SEX==`sex' & CNT2=="`cnt'", barw(`bw') col("48 122
133") lcol(`lc') ///
          || bar DRYN2pos AGEVAR if SEX==`sex' & CNT2=="`cnt'", barw(`bw') col("255
180 0") lcol(`lc') ///
          || bar DRYN3pos AGEVAR if SEX==`sex' & CNT2=="`cnt'", barw(`bw')
col("orange") lcol(`lc') ///
          || bar DRYN4pos AGEVAR if SEX==`sex' & CNT2=="`cnt'", barw(`bw') col("190
190 190") lcol(`lc') ///
      || bar DRYN0neg AGEVAR if SEX==`sex' & CNT2=="`cnt'", barw(`bw') col("0 29
74") lcol(`lc') ///
      || bar DRYN1neg AGEVAR if SEX==`sex' & CNT2=="`cnt'", barw(`bw') col("48 122
133") lcol(`lc') ///
          || bar DRYN2neg AGEVAR if SEX==`sex' & CNT2=="`cnt'", barw(`bw') col("255
180 0") lcol(`lc') ///
          || bar DRYN3neg AGEVAR if SEX==`sex' & CNT2=="`cnt'", barw(`bw')
col("orange") lcol(`lc') ///
          || bar DRYN4neg AGEVAR if SEX==`sex' & CNT2=="`cnt'", barw(`bw') col("190
190 190") lcol(`lc') ///
          || scatter DRYN1 AGEVAR if SEX==`sex' & CNT2=="`cnt'", col(black)  ///
          yline(0, lcol(gs5)) ///
```

```
        graphregion(margin(l-3 r-2 t+0 b-3) color(white)) ///
        plotregion(margin(tiny)) ///
    title("`cnt', men", size(med) margin(tiny)) ///
    ytitle("", size(medsmall)) ///
        ysc(r(`yscl' `yscu')) xsc(r(0.5 3.5)) ///
        ylabel(`ylabl'(5)`ylablu') ///
        xlabel(1 "20-39" 2 "40-59" 3 "60+" 4.6 "Total", labsize(medsmall)) ///
        xtitle("", margin(0 0 0 2)) ///
    legend(off)
    graph display, xsize(`xs') ysize(`ys')
        graph save $graphslocation/dec_`cnt'_`sex', replace
        graph export $graphslocation/dec_`cnt'_`sex'.png, replace

  local sex = 2
  graph twoway bar DRYN0pos AGEVAR if SEX==`sex' & CNT2=="`cnt'", barw(`bw') col("0
29 74") lcol(`lc')  ///
    || bar DRYN1pos AGEVAR if SEX==`sex' & CNT2=="`cnt'", barw(`bw') col("48 122
133") lcol(`lc') ///
        || bar DRYN2pos AGEVAR if SEX==`sex' & CNT2=="`cnt'", barw(`bw') col("255
180 0") lcol(`lc') ///
        || bar DRYN3pos AGEVAR if SEX==`sex' & CNT2=="`cnt'", barw(`bw')
col("orange") lcol(`lc') ///
        || bar DRYN4pos AGEVAR if SEX==`sex' & CNT2=="`cnt'", barw(`bw') col("190
190 190") lcol(`lc') ///
    || bar DRYN0neg AGEVAR if SEX==`sex' & CNT2=="`cnt'", barw(`bw') col("0 29
74") lcol(`lc') ///
    || bar DRYN1neg AGEVAR if SEX==`sex' & CNT2=="`cnt'", barw(`bw') col("48 122
133") lcol(`lc') ///
        || bar DRYN2neg AGEVAR if SEX==`sex' & CNT2=="`cnt'", barw(`bw') col("255
180 0") lcol(`lc') ///
        || bar DRYN3neg AGEVAR if SEX==`sex' & CNT2=="`cnt'", barw(`bw')
col("orange") lcol(`lc') ///
        || bar DRYN4neg AGEVAR if SEX==`sex' & CNT2=="`cnt'", barw(`bw') col("190
190 190") lcol(`lc') ///
        || scatter DRYN1 AGEVAR if SEX==`sex' & CNT2=="`cnt'", col(black)  ///
        yline(0, lcol(gs5)) ///
        graphregion(margin(l-3 r-2 t+0 b-3) color(white)) ///
        plotregion(margin(tiny)) ///
    title("`cnt', women", size(med) margin(tiny)) ///
    ytitle("", size(medsmall)) ///
        ysc(r(`yscl' `yscu')) xsc(r(0.5 3.5)) ///
        ylabel(`ylabl'(5)`ylablu') ///
        xlabel(1 "20-39" 2 "40-59" 3 "60+" 4.6 "Total", labsize(medsmall)) ///
        xtitle("", margin(0 0 0 2)) ///
    legend(off)
    graph display, xsize(`xs') ysize(`ys')
        graph save $graphslocation/dec_`cnt'_`sex', replace
        graph export $graphslocation/dec_`cnt'_`sex'.png, replace

      grc1leg $graphslocation/dec_`cnt'_0.gph $graphslocation/dec_`cnt'_1.gph
```

```
$graphslocation/dec_`cnt'_2.gph, ///
        legendfrom($graphslocation/dec_`cnt'_0.gph) cols(3) ///
        graphregion(margin(l-3 r-2 t+0 b-3) color(white)) ///
        plotregion(margin(tiny))


***********************
* Total
***********************

  /*
        grc1leg $graphslocation/dec_AT_0.gph $graphslocation/dec_EE_0.gph
$graphslocation/dec_EL_0.gph ///
    $graphslocation/dec_ES_0.gph $graphslocation/dec_FR_0.gph
$graphslocation/dec_IT_0.gph ///
    $graphslocation/dec_PL_0.gph $graphslocation/dec_SE_0.gph
$graphslocation/dec_SI_0.gph, ///
        iscale(*0.8) cols(2) ///
        legendfrom($graphslocation/dec_AT_0.gph) ring(0) position(5) ///
        graphregion(margin(l-3 r-2 t+0 b-0) color(white)) ///
        plotregion(margin(tiny))
    graph display, xsize(6) ysize(9)
        graph export $graphslocation/dec_1.eps, replace
  */

        grc1leg $graphslocation/dec_AT_0.gph $graphslocation/dec_AT_1.gph
$graphslocation/dec_AT_2.gph ///
    $graphslocation/dec_FR_0.gph $graphslocation/dec_FR_1.gph
$graphslocation/dec_FR_2.gph ///
    $graphslocation/dec_SI_0.gph $graphslocation/dec_SI_1.gph
$graphslocation/dec_SI_2.gph ///
    $graphslocation/dec_EL_0.gph $graphslocation/dec_EL_1.gph
$graphslocation/dec_EL_2.gph ///
    $graphslocation/dec_ES_0.gph $graphslocation/dec_ES_1.gph
$graphslocation/dec_ES_2.gph, ///
    legendfrom($graphslocation/dec_AT_0.gph) cols(3) ///
        graphregion(margin(l-3 r-2 t+0 b-1) color(white)) ///
        plotregion(margin(tiny))
    graph display, xsize(6) ysize(10)
        graph export $graphslocation/dec_1.eps, replace

        grc1leg $graphslocation/dec_IT_0.gph $graphslocation/dec_IT_1.gph
$graphslocation/dec_IT_2.gph ///
    $graphslocation/dec_EE_0.gph $graphslocation/dec_EE_1.gph
$graphslocation/dec_EE_2.gph ///
    $graphslocation/dec_PL_0.gph $graphslocation/dec_PL_1.gph
$graphslocation/dec_PL_2.gph ///
    $graphslocation/dec_SE_0.gph $graphslocation/dec_SE_1.gph
$graphslocation/dec_SE_2.gph, ///
        legendfrom($graphslocation/dec_IT_0.gph) cols(3) ///
```

```
    graphregion(margin(l-3 r-2 t+0 b-1) color(white)) ///
    plotregion(margin(tiny))
graph display, xsize(6) ysize(9)
    graph export $graphslocation/dec_2.eps, replace
```
